# Genomic RNA folding mediates assembly of human parechovirus

**DOI:** 10.1038/s41467-016-0011-z

**Published:** 2017-02-23

**Authors:** Shabih Shakeel, Eric C. Dykeman, Simon J. White, Ari Ora, Joseph J.B. Cockburn, Sarah J. Butcher, Peter G. Stockley, Reidun Twarock

**Affiliations:** 1 Institute of Biotechnology and Department of Biosciences, University of Helsinki, Helsinki, 00790 Finland; 2 Departments of Mathematics and Biology and York Centre for Complex Systems Analysis, University of York, York, YO10 5DD UK; 3 Astbury Centre for Structural Molecular Biology, University of Leeds, Leeds, LS2 9JT UK; 4 Department of Applied Physics and Department of Biotechnology and Chemical Technology, Aalto University, Espoo, 02150 Finland

## Abstract

Assembly of the major viral pathogens of the *Picornaviridae* family is poorly understood. Human parechovirus 1 is an example of such viruses that contains 60 short regions of ordered RNA density making identical contacts with the protein shell. We show here via a combination of RNA-based systematic evolution of ligands by exponential enrichment, bioinformatics analysis and reverse genetics that these RNA segments are bound to the coat proteins in a sequence-specific manner. Disruption of either the RNA coat protein recognition motif or its contact amino acid residues is deleterious for viral assembly. The data are consistent with RNA packaging signals playing essential roles in virion assembly. Their binding sites on the coat proteins are evolutionarily conserved across the *Parechovirus* genus, suggesting that they represent potential broad-spectrum anti-viral targets.

## Abstract (web summary)

The mechanism underlying packaging of genomic RNA into viral particles is not well understood for human parechoviruses. Here the authors identify short RNA motifs in the parechovirus genome that bind capsid proteins, providing approximately 60 specific interactions for virion assembly.

## Introduction

Picornaviruses are one of the largest groups of positive-sense single-stranded (ss) RNA viruses. They are major pathogens in all kingdoms of life, including human and animal viruses such as polio, human rhinovirus, and foot and mouth disease virus. Their virion assembly mechanism(s) has been extensively studied. Previous results suggest that they are an exception to the general findings in other examples of RNA viruses, that typically appear to exploit at least one high affinity coat protein (CP) binding site within their genomes to initiate virion assembly. In contrast, many studies including extensive nucleotide recoding of large sections of the polio genome have failed to identify any RNA determinants involved in picornavirus assembly^1–3^. Multiple X-ray crystallographic and electron microscopic three-dimensional picornavirus structures show very little ordered density for the encapsidated nucleic acid, which would be consistent with the RNA playing no role in virion assembly.

Recently, starting with the medium resolution EM reconstruction of human parechovirus 1 (HPeV1), several parechovirus structures have been reported that do contain extensive density for RNA in close contact with the overlying protein capsid, amounting in some cases to over 10% of the genome^4–8^. All these virions contain 60 such RNA fragments that make identical contacts with their CPs around the five-fold vertices. The occupancy levels of the density are high, suggesting that they reflect important aspects of the virion structure. All of these viruses differs from the majority of picornaviruses in that they do not cleave their VP0 polyproteins into VP2 and VP4. This may be related to the presence of ordered RNA density. The role(s) of this RNA density, however, requires a functional explanation, especially as these structures may represent an earlier evolutionary state of the wider group of picornaviruses. The assembly and positioning of defined RNA fragments at precise locations within these virions suggests that their genomes may play an important role(s) in virus assembly.

Previously we identified, in plant and bacterial viruses, an assembly mechanism we have termed packaging signal (PS)-mediated assembly^9–13^. The classical PS hypothesis assumes the presence of a single PS site (a specific RNA fragment) with affinity for cognate CP that results in formation of an RNA-CP assembly initiation complex. PS-mediated assembly differs from this by recognising that multiple sites across ssRNA viral genomes can act as PSs, although these sites vary around a consensus sequence and thus have widely varying intrinsic affinities for CP^14,15^. Indeed, mathematical modelling of such assembly mechanisms suggests that modulation of CP-PS affinity is an essential feature of efficient assembly in order to avoid kinetic trapping^16^. Sequence variability in the PSs are therefore expected. Due to this, PS sites cannot be identified by a simple bioinformatics search for a repeating sequence motif.

Here, we identified multiple PSs dispersed throughout the HPeV1 RNA genome using RNA-based systematic evolution of ligands by exponential enrichment (RNA SELEX), bioinformatics analysis and mutational analysis of an infectious HPeV1 clone. These PSs are conserved throughout the *Parechovirus* genus. The sequence-specific PS-CP contacts that we identified could be potential targets for a novel broad-spectrum anti-viral therapy. Our insights into the processes of genome encapsidation and virion assembly may also be useful in the development of stable vaccine candidates.

## Results

### Multiple HPeV1 genome regions have affinity for CP

**Fig. 1:**
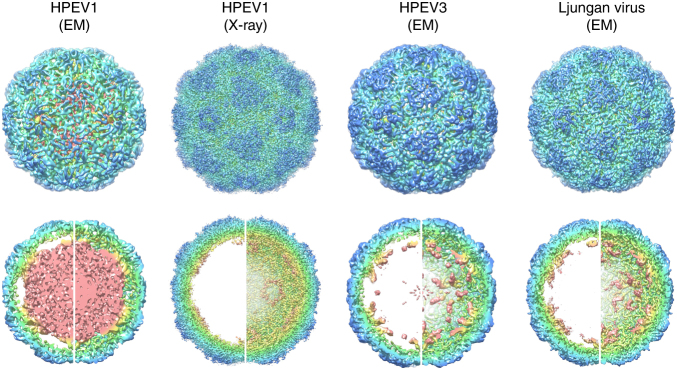
**Ordered RNA segments in the structures of picornaviruses.** Four icosahedrally-symmetric structures for parechovirus virions are currently available. These are HPeV1 (Cryo-EM, 8.5 Å resolution, EMD-1690)^7^, HPeV1 (X-ray crystallography, 3.1 Å resolution, PDB 4Z92)^4^, HPeV3 (Cryo-EM, 4.3 Å resolution, EMD-3137)^8^, and Ljungan virus (Cryo-EM, 4.5 Å resolution, EMD-6395)^5^. On the *top row*, an exterior view of each capsid is shown, viewed perpendicular to an icosahedral two-fold axis. On the *bottom row*, each virus is shown in the same orientation, as a 60 Å thick central section on the left hand side, and the rear half of the capsid on the right hand side. All panels are coloured with an identical radial colour scheme (*red*: 98 Å, *yellow*: 111 Å, *green*: 124 Å, *cyan*: 137 Å, *blue*: 151 Å). The diameters of all capsids are very similar, with density ascribed to RNA shown in *yellow*/*red* in each case. More RNAs can be seen in the lower resolution HPeV1 EM reconstruction at 8.5 Å, than in the X-ray density at 3.1 Å resolution, suggesting that only a few nucleotides are identical in all 60 positions, but similar stem-loops occupy all positions

**Fig. 2:**
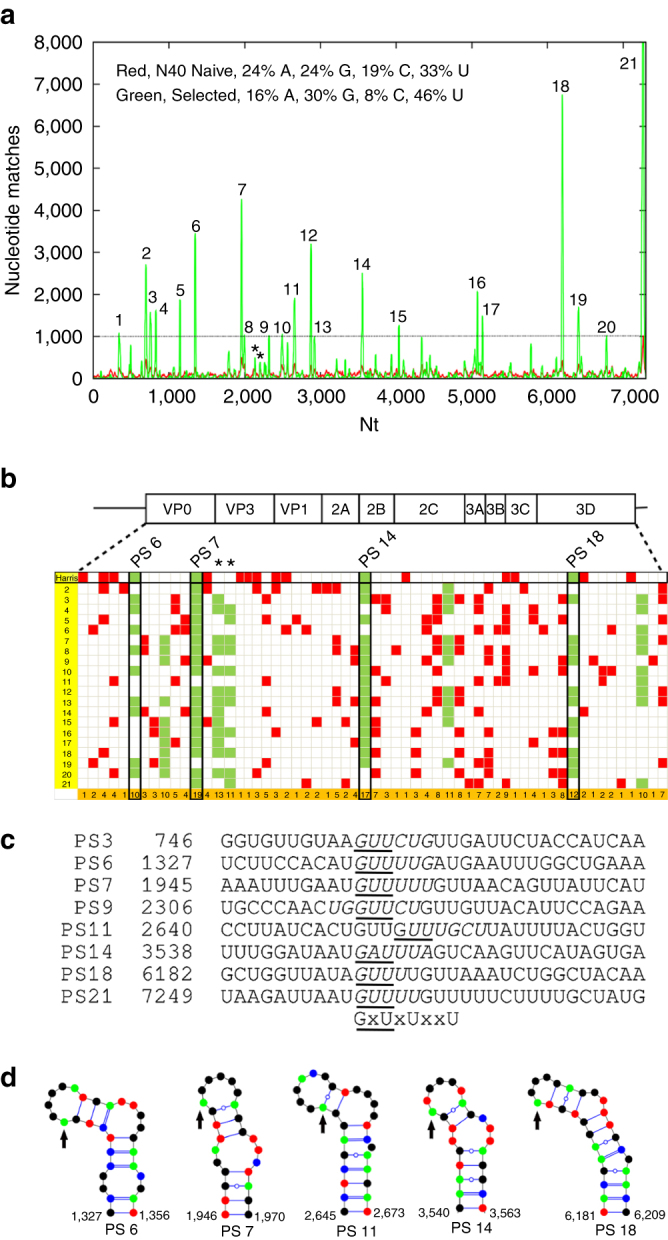
**Identification of putative PS positions within the HPeV1 Harris genome.** **a** Histogram plot showing alignments of aptamer sequences with Bernoulli scores ≥ 12 to the HPeV1 genome for the enriched SELEX pool (*green*) and the N40 naïve library (*red*), base compositions of both pools shown *inset*. The *dashed line*, corresponding to the highest peak in the match of the naïve library to the Harris genome, indicates the initial threshold used to identify areas with statistically significant sequence similarities. **b** Alignment of the results of similar plots for the other 20 complete genome sequences of HPeV1 strain variants are shown with the data from the Harris strain (vertical axis, Harris strain = 1; see Supplementary Table 1 for the Genbank IDs). The positions of statistically significant peaks, i.e. peaks above the level of the highest background peak, within the coding regions of each genome are shown as filled cells in the Table. These were aligned within the polyprotein coding region using Clustal^19^. Peaks within the Harris strain are termed PSs and are aligned with equivalent sites in the other strains, provided the peak nucleotides lie within 10 nucleotides of the Harris PSs. The last row of the table shows the number of such matches from all the strains. Co-localised peaks in more than 47% of the strains are shown in *green*, the others are in *red*. The asterisks in **a**, **b** indicate two peaks, PS8A and PS8B, which are below the statistical cut-off defined by the naïve library but are highly conserved in >47% of the non-Harris strains. They have similar predicted secondary structures, suggesting that they are also PSs (Supplementary Fig. 2b). **c** Alignments of a subgroup of Harris PS secondary structures, some of which are shown as VARNA^39^ representations in **d** with G—*green*; C—*blue*; A—*red* and U—*black*. Sequences that appear in the terminal loops of these structures are italicised in **c** while occurrences of the triplet GxU (see Fig. 3) are underlined. **d** Mfold structures for four PSs with the *arrows* indicating the start of the GxU motifs that occur at the 5′ end of the single-stranded loops (see Supplementary Fig. 2)

Inspired by our first picornavirus structure, showing extensively ordered RNA density^7^ (Fig. 1), we used bioinformatics of RNA SELEX outputs to identify preferred RNA binding motifs. Pentameric capsomers of VP0, VP1 and VP3 are widely assumed to be the basic building blocks of picornavirus capsids^17,18^. We therefore identified conditions (heating to 56 °C for 30 min, followed by incubation at 4 °C for 10 days) under which we could reproducibly generate CP pentamers from purified HPeV1 virions (Harris strain), i.e. pentamers that are assembly competent (Supplementary Fig. 1). RNA SELEX was then used to isolate preferred binding sequences for these pentamers immobilised on magnetic beads (see Methods, and Supplementary Fig. 1). Counter-selection against intact virions was used to remove aptamers with affinity for the exterior surface of the virus, thus biasing the aptamers isolated to those that bind to the interior surface of the capsid where the ordered genome segments are seen. The RNA library used for SELEX encompasses a 40 nucleotide region in which every position is randomized. Fragments of this size exceed the likely RNA content of the ordered EM density (Fig. 1), allowing us to have complete coverage of likely binding motifs. The 10th round of selected aptamers was converted to cDNA and subjected to Next Generation DNA Sequencing. This yielded ~600,000 aptamer sequences, of which ~400,000 were unique from an initial starting library of ~10^15^ sequences. Individual aptamers in the selected pool occur between 100–10,000 times, compared to at most one or two occurrences of each sequence in the unselected (naïve) library. This outcome, together with the change in nucleotide composition of the starting and final aptamer pools (see inset in Fig. 2a), confirmed that efficient selection of pentamer binding aptamers had been achieved.

If the genomic segments in contact with the CPs in the virions were bound sequence-specifically, we would expect these aptamers to share some primary/secondary structure motifs with them. Since the minimal CP recognition motifs within a PS may be discontinuous and/or structurally degenerated, e.g. by differing in the sequences of non-contacted base pairs, the number of identical nucleotide positions might be quite low. Therefore, in order to identify putative genomic sites with affinity for CP, we aligned all the aptamer sequences against every position in the Harris genome using a 1 nt sliding window. We quantified the goodness of any match between an aptamer and the genomic sequence with reference to the probability equivalent of finding a contiguous match. For example, if a non-contiguous match across a length of N nucleotides has the same probability as a contiguous match across M < N nucleotides, then we used M, known as the Bernoulli score (see Methods), to quantify the goodness of fit of the alignment. Since alignments of 12 nucleotides in a row are rare (a probability of about 1 in 10,000), we identified all alignments with a Bernoulli score of at least 12. For each such aptamer, we increased a counter by one at every matching nucleotide position, resulting in the histogram plot in Fig. 2a, which corresponds to the summation of the counters in each case for the aptamer pool (*green* curve) and naïve library (*red* curve). Roughly 6% of the selected unique aptamer sequences match the genome with this stringency. The frequency of matches within the selected aptamer pool is much larger than for the naïve library, with one exception, a polyU tract towards the 3′ end of the RNA. For aptamers corresponding to peaks below the level of this highest background peak, SELEX has not resulted in a sufficient enrichment to conclude a priori that the matching sites in the genome sequence are more significant than peaks resulting from an alignment of the naïve library. We therefore initially excluded these sites from analysis, leaving 21 statistically significant peaks dispersed across the genome. This result suggests that there are sequences within the HPeV1 genome that are likely to show specific affinity for the overlying CP, and these in turn are candidates to correspond to the ordered segments seen in the virion structure.

The variation in PS-CP affinities in other viral systems is manifest in subtle variations in the PS sequences/structures across the genome, making identification of PS recognition motifs based on data from a single virus genome challenging. However, due to their functional importance in assembly, PS sites are potentially conserved across strain variants. We therefore used all 21 complete HPeV1 genome sequences available from GenBank (see Supplementary Table 1 for strain IDs) to explore whether this is indeed the case here. For this, we carried out the same analysis as in Fig. 2a for all strain variants, revealing similar statistically significant peaks in those genomes, i.e., peaks above the level of the highest background peak. In order to compare those peaks, which correspond to the putative PS positions across all the variant strains, we aligned the RNA sequences with Clustal^19^ using the polyprotein gene region as a constraint. Whenever peak positions were located within at most 10 nt from each other in this alignment, we grouped them. Such peak positions are represented by columns in Fig. 2b, with cells representing the different strain variants in a group highlighted. Groups with peaks in <47% of all strains are indicated as red cells. Where >47% of all strains have a peak at the same position, all peaks in that column are shown as green cells to emphasise their higher level of conservation.

This level of conservation suggests that the genomic sequence matches correspond to RNA packaging signals (PSs). Such PSs would be expected to encompass a common CP recognition motif. To explore this possibility, we extracted 41 nt long fragments around each of the highly conserved peaks from the Harris strain (PS6, PS7, PS14, PS18), two higher peaks but with low conservation (PS3, PS11), a lower peak close to the statistical cut-off (PS9), and the highest peak in the non-coding regions (PS21), each containing 20 nt of the genomic sequence on either side of each peak nucleotide in the histogram plot (see second column in Fig. 2c for peak positions). Alignment of these fragments reveals a conserved GxUxUxxU motif in the central area of these fragments (Fig. 2c). We checked using Mfold^20^, that there are secondary structure folds for each of the selected PSs, except PS11, encompassing at least six out of the eight nucleotides in the loops of a stem-loop (Fig. 2d). PS11 forms an exception in that only four nucleotides of the identified GxUxUxxU motif overlap with the loop of the stem-loop. However, this is due to the repeat occurrence of the GxU motif, which could not be properly positioned based on sequence information alone. Indeed, taking both sequence and structure into account, the second GUU (underlined in Fig. 2c) should be aligned with the GxU consensus motif. In this case, also for PS11, six nucleotides overlap with the loop of the stem-loop. However, this implies that the second U in the consensus motif may be less important than the start GxU motif. This is corroborated further by augmenting the alignment with the sequences corresponding to other PSs in the ensemble, which shows that indeed the GxU triplet at the 5′ end is the core part of the consensus motif in Fig. 2c. In every case in Fig. 2d, the folds either include the GxU triplet at the 5′ end of the motif, or do so after opening of the G-U pair at the bottom of the loop as in the case of PS11, suggesting again that those nucleotides are functionally more important. We then analysed the folds of the sequences corresponding to all other peaks in the Harris strain above the cut-off. These all present the shorter, GxU motif in the loop, or in three cases, this motif would be single-stranded if a single base pair at the bottom of the loop is broken (Supplementary Fig. 2).

For genomic sites corresponding to an additional 52 Bernoulli peaks below the cut-off, but that are higher in the selected library than in the naïve one, the SELEX procedure has enriched the pool of matching aptamers. In competition with other aptamers, these have performed less well in CP binding, but they could still be associated with stem-loops containing the GxU motif and thus correspond to PSs. As before, we extracted 41 nucleotide fragments around the peak nucleotide for each of these 52 peaks, and established that 50 of these can fold into stem-loops containing a GxU motif in the loop. This thus identified 71 of the 73 Bernoulli peaks above the lower cut-off criterion as putative PSs. Two peaks (termed PS8A and B), each marked by an asterisk in Fig. 2a, are of interest, because they are highly conserved as significant peaks in >50% of the strains other than Harris (Fig. 2b). Inspection of their potential secondary structures (Supplementary Fig. 2b) again reveals the conserved sequence motif, suggesting that these additional putative PS positions below the statistical cut-off also correspond to PSs. The occurrence of 71 Bernoulli peaks in total with the characteristic recognition motif suggests that there is a sufficient number of putative PSs to occupy the 60 positions of high RNA density in contact with CP in the virion structure (Fig. 1). Evaluation of the lowest free energy Mfold secondary structures for the selected aptamer library of ~10^5^ unique sequences shows that ~77% of them fold into stem-loops displaying a GxU motif within the loop. This result confirms that aptamer selection was dominated by this feature.

### The motif is bound sequence-specifically in the virion

The recent HPeV1 X-ray structure at 3.1 Å resolution allows us to answer the question of whether these putative PSs correspond to the ordered RNA segments seen in the virion, and whether they bind to the CP in a sequence-specific fashion^4^. High-resolution icosahedral averaging often results in the loss of RNA density, because the genome is an inherently asymmetric object with a unique nucleotide sequence. The extent of ordered RNA in the X-ray map is thus significantly less than that in the EM map (Fig. 1), but Kalynych et al. could build a model for a 6 nt long RNA (AUUUUU) in the form of an extended single-strand contacting the protein shell.

**Fig. 3:**
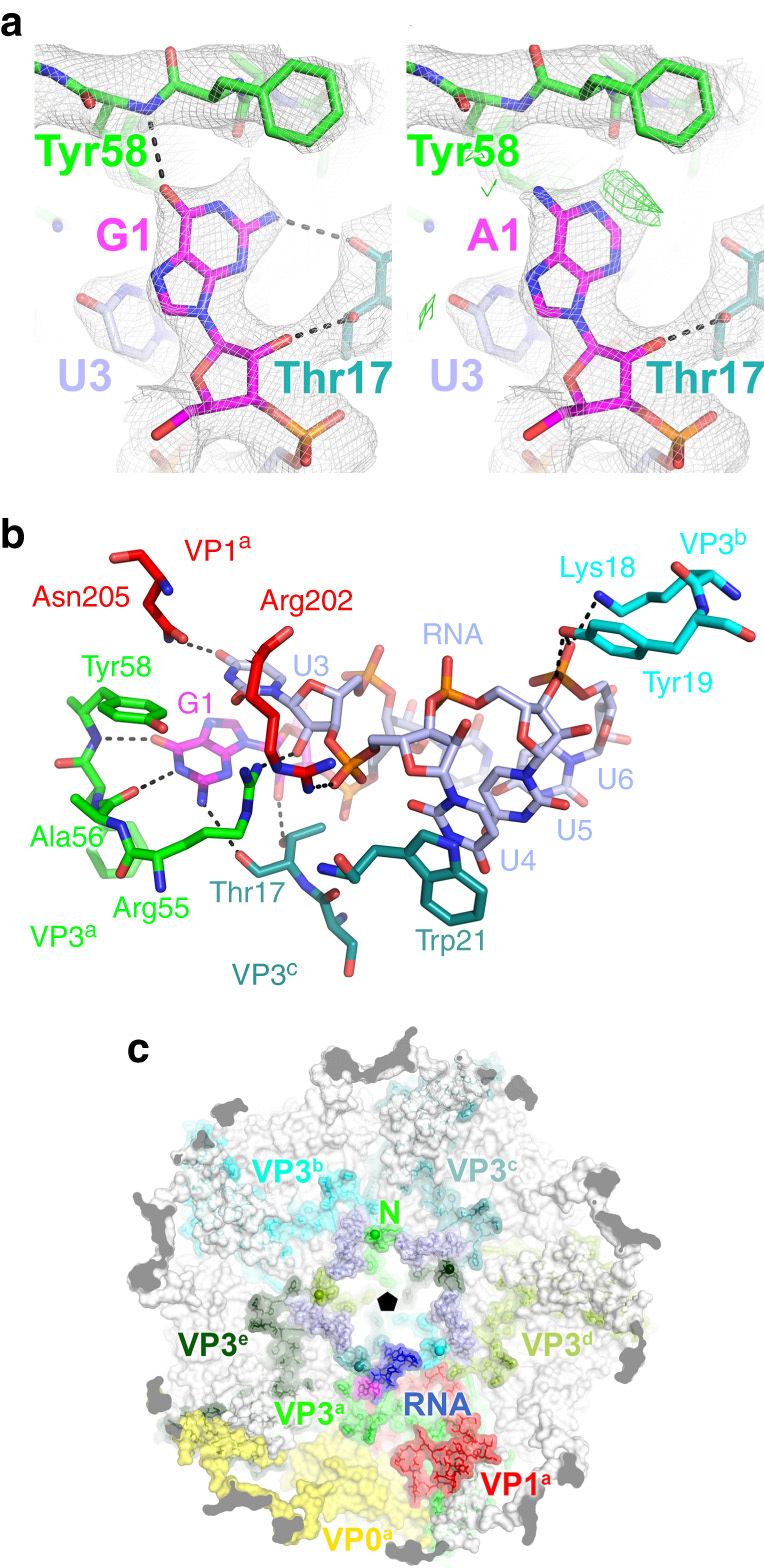
**HPeV1 protein:RNA interactions.** A recently published crystal structure by Kalnych et al., PDB: 4Z92, had a new model (PDB: 5MJV) built into the electron density^4^. **a** Comparison of the refined crystal structures of the virus with guanosine (*left*), and adenosine (*right*) modelled in position 1 of the RNA. Sigma-weighted 2mF_o_–DF_c_ maps are contoured in grey at 1.0σ. In the *right* panel, the mF_o_–DF_c_ difference map is contoured in *green* at 2.5σ. **b** Close-up showing a single copy of the viral RNA and its interactions with the surrounding amino acid residues from the viral CPs. **a**, **b** Protein and RNA are shown in stick representation with the following colours: oxygen, *coral*; nitrogen, *dark blue*; phosphorus, *orange*. Carbon atoms are coloured by subunit as shown in **b**, **c**; additionally, carbon atoms in the RNA base G1 are shown in *magenta*. Putative hydrogen bonds shown as *dashed lines*. **c** View of the viral capsid from inside the virus showing the arrangement of proteins and viral RNA around the icosahedral five-fold. VP1, VP3 and VP0 from one asymmetric unit (denoted VP1^a^, VP3^a^ and VP0^a^) are shown in *red*, *green* and *yellow* respectively. VP3 contains a long N-terminal extension that reaches around the icosahedral five-fold. The N-terminal residue of each copy of VP3 in the crystal structure (Met15) is marked with a ball on the nitrogen atom and labelled N for VP3^a^. The RNA associated with VP1^a^ and VP3^a^ is shown in *dark blue*, with the 5′ guanosine in magenta. Five-fold related copies of the RNA are shown in *light blue*. Five-fold related copies of VP3^a^ (VP3^b^ through VP3^e^) are also coloured in different shades of *green* and labelled as shown. Note how the VP3 N-terminal regions interdigitate between adjacent RNA copies around the five-fold, so that each RNA is recognised by VP1 and 3 copies of VP3 (VP3^a^, VP3^b^ and VP3^c^ for the RNA molecule shown in *dark blue*, see Table 1)

When we examined the deposited electron density map, we found that some of these RNA residues were poorly modelled (Fig. 3a). We therefore rebuilt and re-refined the structure, adding an atomic model of the PS consensus into the resulting density for RNA yielding a structure with an improved *R*_free_ factor (0.26). The model is an excellent fit (Fig. 3a) and reveals extensive RNA-CP contacts (Table 1 and Supplementary Table 2), comprising both hydrogen bond as well as van der Waals contacts. The RNA contacts multiple CP, principally VP3 and VP1. The contacts include three base-specific hydrogen bonds, two to the initial purine, which is therefore confirmed as the consensus G rather than the previously modelled A, although the latter could also be accommodated in this position. There is also a uridine-specific contact to the base N+2 3′ to the G (Fig. 3b). Most nucleotide bases are orientated away from the protein, consistent with the SELEX result. N+3 is modelled as a uridine which stacks on the side chain of Tyr21, although any base could make this interaction. The neighbouring N+4 position is fixed as U by SELEX and participates in an extended stacking interaction with U4, although why it needs to be uridine is unclear. The remaining fixed nucleotide in the SELEX consensus is outside the X-ray density. The fact that not all PSs contain these latter two fixed bases may indicate that their identities are less critical.

**Table 1:** Potential hydrogen bonds between RNA and capsid

RNA			Protein				Distance (Å)
Residue	Identity	Atom	Subunit	Residue	Identity	Atom	
1	G	N1	VP3^a^	56	Ala	O	3.1
1	G	O6	VP3^a^	58	Tyr	N	3.0
1	G	O2′	VP3^c^	17	Thr	OG1	2.85
1	G	N2	VP3^c^	17	Thr	O	3.31
3	U	O4	VP1^a^	205	Asn	ND2	3.19
3	U	O2′	VP3^a^	55	Arg	NH2	2.64
4	U	OP2	VP1^a^	202	Arg	NH2	2.56
5	U	O3′	VP3^b^	19	Tyr	OH	2.97
6	U	OP1	VP3^b^	18	Lys	NZ	2.83
6	U	OP1	VP3^b^	19	Tyr	OH	3.29

^a,b,c^refer to the proteins belonging to different asymmetric units in the pentamer, as shown in Fig. 3

Amino acid residues in each extended VP3 arm make multiple backbone hydrogen bonds to the RNA fragment neighbouring the one that they contact sequence-specifically, creating a chain of RNA-CP contacts surrounding each five-fold axis (Fig. 3c). This arrangement strongly suggests that the capsomer for PS binding is a pentamer, because these important recognition sites would not be present on a protomer. In addition, each region of RNA buries ~1,120 Å^2^ at the interface of the CPs and each PS, i.e. 5,600 Å^2^/capsomer and 67,200 Å^2^/capsid. This is a very large surface area and could easily provide the binding free energy to drive capsid formation and the confinement of the polynucleotide genome.

These base-specific contacts match precisely the motif GxU derived from SELEX and bioinformatics, confirming that this approach has identified the consensus of the ordered RNA fragments in the virion in striking detail. Thus, the X-ray structure confirms the recognition motif. A close up of the extended EM density for one of the ordered RNA fragments is consistent with the recognition motif being presented on a stem-loop structure (Supplementary Fig. 3). Binding of the GxU motif within the site shown in Fig. 3, however, requires an element of induced fit since the upper stems of the secondary structures shown in Fig. 2 and elsewhere must become single-stranded to allow this to occur. There are 436 copies of the GxU triplet within the HPeV1 Harris genome, 185 of which occur as part of non-overlapping segments of the form GxUxxx flanked by 10 nucleotides either side. By comparison, only 71 of the GxU motifs appear in the loops of stem-loop structures above background in Fig. 2a. If the triplet was recognised during virus assembly by sliding the genome sequence against the pentamer, there would be no defined secondary structure in the flanking regions and no ordered density beyond the hexanucleotide. The EM data show that this is not the case.

### The conserved PSs play roles in virion assembly

To test whether the bound RNA fragments are important for virion assembly, we probed their functions in vivo. Reverse genetics was used to create mutational variants (PS#-M) of a number of the individual PS sites in the context of an infectious HPeV1 cDNA clone (wild-type, WT) under the control of a T7 promoter. We introduced the minimal number of silent mutations required to disrupt the potential secondary structure of each chosen PS separately. In order to assess whether the peak heights in the Bernoulli plot (Fig. 2a) reflect the relative importance of individual PS sites, we chose PSs within both the coding and non-coding regions corresponding to different peak heights, namely PS3, PS9, PS11 and PS21. We also altered the highly conserved PSs in the coding region (PS6, PS7, PS14 and PS18).

To test the effects of these mutations on infectious virus production, linear cDNA encoding each mutational change was transfected into a Green Monkey Kidney (GMK) cell line transiently expressing T7 RNA polymerase that then transcribes the viral genome. The viral titres in each case were determined by preparing cell lysates, treating them with RNase and DNase and then assaying their titres on HT29 cells using the 50% tissue culture infectious dose (TCID50) method. HT29 cells were chosen as they give a clear cytopathic effect with TCID50/ml as low as 10^1^ being unambiguously detected. In order to exclude the possibility that the outcomes are the result of effects on replication, we confirmed that the wild-type and mutants produced similar amounts of viral RNA using real-time PCR. Further, immunofluorescence with an anti-dsRNA antibody showed that there are also similar numbers of viral replication centres for all mutants in all the cells used. All the mutant genomes tested (except PS9-M) also produce similar in vitro translation products. Hence, in PS21, PS14 and PS18, the decreases in viral titre are most likely the result of alterations in assembly efficiency (Table 2 and Supplementary Fig. 4).

**Table 2:** PS mutants tested in cell culture and in vitro

**Virus variant (genetic locus)**	**Nucleotide locations (see** Fig. 2a/b **)**	**Sequences of the tested mutated PS** ^**a**^ **(5′–3′)**	**50**% **Tissue culture infectious dose (TCID)/ml** ^**b**^	**Replication** ^**c**^	**Translation** ^**d**^
Wild-type	N/A	N/A	10^6^	Yes	Yes
PS3-M (VP0)	745–776	UGGUGUUGUcA*Gcagc* GUUGAUagcACCAUCA	10^6^	Yes	Yes
PS6-M (VP0)	1310–1360	UUcGGAGCuUUUACgAAcCUUCCACAU*GUUcUu*AUGAAccUGGCUGAAACC	10^6^	Yes	Yes
PS7-M (VP3)	1931–1981	cUgAGACUgUUcCCAAAcUUGAAU*GU* *gUU* *U*GUgAACAGUUAcaguUACUUU	10^6^	Yes	Yes
PS9-M (VP3)	2304–2336	UCUGCCCAAC*UGGaagU*GUaGUaACAUUCCAGA	0	Yes	No
PS11-M (VP1)	2645–2673	UCACUacUc*UUcGCc*UAcUUcACUGGUGA	10^6^	Yes	Yes
PS14-M (2B)	3524–3571	GCcGCgACcGAGAUUcUuGAUAAc*GAU* *cUc*GUCAAGUUCAUAGUGAAA	10^4^	Yes	Yes
PS18-M (3D)	6181–6209	AGCcGGaUAcu*ccUU*cGUcAAAagcGGCU	10^5^	Yes	Yes
PS21-M (3ʹ UTR)	7250–7276	UAAGAcgAAU*Gaaac* GUUcgUCUUUUG	0	Yes	Yes

^a^The nucleotides substituted within each mutant PS are in lower case, the loop region of each PS is italicised and the GxU motifs are underlined

^b^Values are the mean TCID50 values calculated from two independent experiments

^c^Replication efficiency of each mutant was tested by staining with anti-dsRNA antibody and by quantitative real time PCR

^d^Translation efficiency of each mutant was tested by phosphorimaging of in vitro translation of mutants labelled with ^35^S-methionine

PS9-M and PS21-M each result in 6-log drops in TCID50 with no cytopathic effect (Table 2), while PS14-M and PS18-M show less dramatic reductions in viral fitness (2 and 1-log drops, respectively). There are no effects for PS3-M, PS6-M and PS7-M. The variation in mutational effects is striking, and may be the result of the way the virion is assembled (see below). It is entirely consistent with previous modelling^14,16^ with the differing impacts of mutating individual PSs on the overall assembly efficiency being expected in the context of a co-operative process. Given the number of individual putative PS sites that appear to contribute to viral assembly, this is strong evidence in favour of PS-mediated assembly in which multiple PS sites act collectively.

In addition to testing PS mutants, we also made single alanine mutants of the individual capsid protein residues in contact with the RNA^4^. All the mutants except VP3 T17A showed 6-log decreases in TCID-50 with no cytopathic effect, but have faithful replication and translation (Table 3 and Supplementary Fig. 5). VP3 T17A behaved like the WT, perhaps because the mutation was relatively conservative, or its effect may be compensated by interaction of the nearby Y19 with the RNA.

**Table 3:** Capsid proteins VP1 and VP3 alanine mutants tested in cell culture and in vitro

Virus variant (genetic locus)	Amino acid location	50% Tissue culture infectious dose (TCID)/ml^a^	Replication^b^	Translation^c^
Wild-type	N/A	10^6^	Yes	Yes
VP1	R202A	0	Yes	Yes
VP1	C203A	0	Yes	Yes
VP3	T17A	10^6^	Yes	Yes
VP3	Y19A	0	Yes	Yes
VP3	W21A	0	Yes	Yes
VP3	T44A	0	Yes	Yes
VP3	R55A	0	Yes	Yes
VP3	R68A	0	Yes	Yes

^a^Values are the mean TCID50 values calculated from two independent experiments

^b^Replication efficiency of each mutant was tested by staining with anti-dsRNA antibody and by quantitative real time PCR

^c^Translation efficiency of each mutant was tested by phosphorimaging of in vitro translation of mutants labelled with ^35^S-methionine

The capsid protein and PSs mutational results confirm the specificity of these capsid protein-RNA interactions, and further consolidate our hypothesis that specific PS-CP amino acid interactions facilitate HPeV1 assembly.

The detailed molecular mechanisms of PS action can vary from virus to virus. In at least one case, they overcome repulsive electrostatic contacts between protomers in a capsomer^13^, and in another, they act as allosteric effectors to create the quasi-equivalent conformers required to assemble capsids of the correct size and symmetry^21,22^. In each of these examples, individual PSs aid the formation of capsomers of the geometry required for capsid formation, albeit via different specific modes of action (e.g. allosteric switching or bridging electrostatic barriers), hinting at the spectrum of effects PSs may have on assembly. Here, stabilisation of the HPeV1 virion is very likely to be one such PS function.

**Fig. 4:**
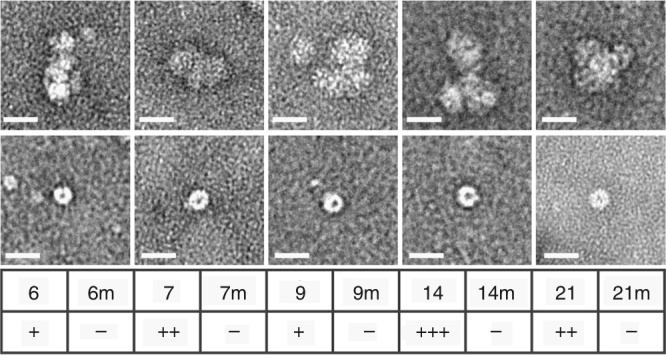
**In vitro pentamer binding to the predicted PSs.** In vitro analysis of PS: pentamer interactions by MST, HPeV1 pentamers were titrated into fixed amounts of 5ʹ-Alexa 488-labelled short RNAs encompassing PSs 6, 7, 9, 14 and 21 and their variants (labelled m). The binding data were assessed semi-quantitatively (+/−) noting the amplitude of the signal change over the range of pentamer concentrations and where significant binding began to occur. These were compared to a known PS-CP interaction, that of the 19 nt bacteriophage MS2 TR (translational operator) stem-loop and its CP^40^ (*K*_*d*_~1–4 nM, depending on binding assay used), which was designated as +++++, i.e. low nano-molar affinity. The highest protein concentration samples from these assays were recovered, negatively-stained and examined by electron microscopy (*top row* shows the PS and the *bottom row* shows its mutant). Scale bar is 25 nm

To assess other possible effects we carried out in vitro binding assays to characterize the interactions of oligonucleotides encompassing some of the individual PS sites and their mutated versions, with CP pentamers. This is challenging because of the small amounts of the CP readily available (<200 µg). In order to conserve the CP, we carried out microscale thermophoresis (MST) binding assays with fluorescent dye end-labelled RNA PSs (Methods). MST is a non-destructive technique that can be carried out in a volume of ~10 µl, allowing a CP titration to be performed into a fixed amount of RNA. All the PSs showed binding that can be roughly rank ordered by affinities as PS14 > PS7/21 > PS6/9 (PSs 3, 11 and 18 were not tested). In contrast, all the associated variant sites did not bind under these conditions, suggesting that the mutational changes introduced had ablated CP recognition both in this assay and presumably in vivo in the context of the intact genome. Transmission electron microscopy of the highest MST titration points suggested that the cognate PS oligos, but not their mutational variants, trigger a partial self-association reaction (Fig. 4). The micrographs show the sequence-specific formation of pentameric aggregates, but not identifiable partial or complete virus-like particles (VLPs). One possibility is that well-defined higher order assemblies do not form, because in the presence of short oligonucleotides, there are no RNA connections possible between pentamers. We therefore repeated the EM assembly assay for unlabelled, protein-free full-length genomic RNA. No VLP formation was detectable. Although the assays used are not very sensitive, they imply that CP and RNA sequence alone are insufficient to regulate assembly.

**Fig. 5:**
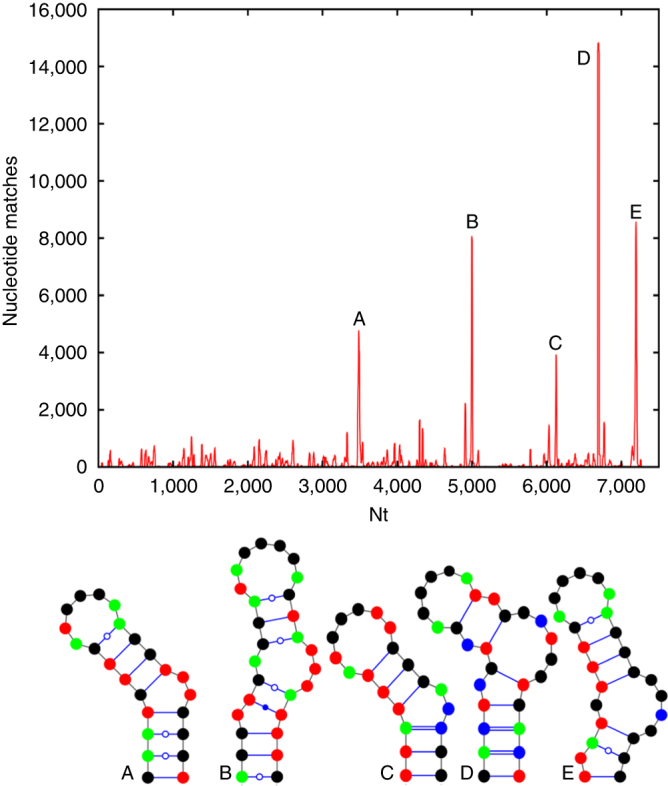
**Conservation across the HPeV genus.** We used the selected HPeV1 library to derive a Bernoulli plot for a representative of the HPeV3 group (GQ183026), which is most distal from the HPeV1 group from the point of view of VP1-VP4 conservation (Supplementary Fig. 6). The number of nucleotide matches of the HPeV3 genome to the selected HPeV1 library is remarkable: with up to 15,000 nucleotide matches for higher peaks and smaller peaks between 1000 and 2000, as opposed to a maximum of 8000 for the higher peaks and 1000–3000 for the lower peaks in HPeV1, indicating that a similar recognition motif is likely to occur also in HPeV3. Consistent with this, sequence fragments ±20 nucleotides around the peak nucleotides can fold into secondary structures that reveal the characteristic GxU motif in the loop of a stem-loop

Following the same procedure used earlier for the Harris strain, we moreover established that in all 45 HPeV sequences available from GenBank (spanning groups 1 to 8, see Supplementary Table 3), there are between 76 and 96 occurrences of non-overlapping stem-loops with the HPeV1 recognition motif (a GxU motif in the single-stranded loop portion of a stem-loop). The numbers are consistent with the 60 potential RNA-binding sites seen in Fig. 1. A phylogenetic analysis of the VP0 polyprotein sequences of all 45 HPeV strains using SplitsTree^23^ identifies HPeV3 (*green arrow* in Supplementary Fig. 6) as most distal from HPeV1 (*red arrow* in Supplementary Fig. 6). Given the high degree of conservation of the binding sites, we used the selected aptamer library of HPeV1 to identify statistically significant matches with a representative of the HPeV3 group (accession no. GQ183026) (Fig. 5). We established that the highest Bernoulli peaks, labelled Peak A to Peak E in order of occurrence in that genome from the 5′ to the 3′-end, indeed correspond to sequence/secondary structure folds akin to the HPeV1 PSs identified in this paper. They also correspond to peaks at defined positions within the genome, namely peaks 14, 16, 18, 20 and 21 when compared with the Bernoulli plot for HPeV1 (Fig. 2a). We also searched the Ljungan virus genome for the characteristic PS motif/structure, and found 95 such stem-loops, suggesting that this recognition motif could be important also for other picornaviruses. This strongly suggests a high degree of PS conservation in the *Parechovirus* genus.

## Discussion

These data support the idea that multiple RNA-based PSs are present in this genus of picornaviruses, and that they each contribute differentially to assembly efficiency, as expected for a PS-mediated process. This idea contrasts with more classical ideas of a single PS per genome at which capsid assembly would initiate.

Our results highlight that up to 60 PSs are presented in spatially-defined sites created by the CPs and that these PSs may be folded as stem-loop structures. In the related Ljungan virus, it was suggested that electrostatic contacts predominate in assembly^5^. However, although interactions are inevitable in forming protein-nucleic acid complexes, they do not necessarily contribute the majority of the binding energy^24^. Our in vitro assembly results with full length genomic RNA suggests that CP binding does not occur spontaneously, the genome needs to be actively folded into an assembly-competent conformation to facilitate assembly. A biologically-relevant explanation for this comes from consideration of the many roles that genomic RNAs play in the viral lifecycle, amongst which being a packaging substrate is just one. In other viruses that assemble using PS-mediated mechanisms, there are competing RNA conformations that regulate replication and translational efficiency or assembly. Being able to alter the global RNA conformation in response to changes in the viral lifecycle is clearly a powerful way to regulate the timing of assembly.

**Fig. 6:**
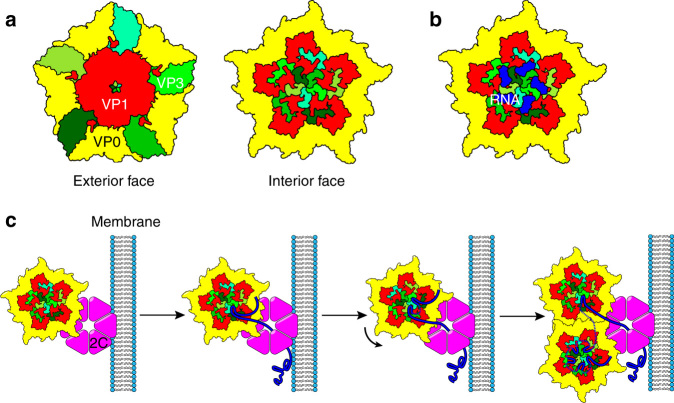
**Cartoon of a plausible virion assembly mechanism.** The crystal structure (PDB: 4Z92)^4^ was used to create a simplified cartoon of the pentameric capsomere. **a** *left*, shows a view along the five-fold axis into the virion, i.e. the outer surface. The CP subunits are coloured, *yellow* for VP0; *red* for VP1 and various shades of *green* for the different VP3 subunits. The *right hand* panel shows the view along the same axis from the centre of the virus, highlighting the extension of the N-terminal arms of the VP3s around the symmetry axis. **b** shows a similar view with the ordered RNA segments in *dark blue*. **c** A cartoon of a hypothesised model of capsid assembly based on known aspects of picornavirus morphogenesis^2^. Pentamers of the viral CPs are bound by the 2C^ATPase^, associated with a replication factory at the cell membrane via sequence-specific protein-protein contacts to VP1 or VP3. Here they contact newly replicated genomic RNA. The 2C^ATPase^ is known to play multiple roles in virion replication and morphogenesis. Its RNA helicase activity would allow it to bias RNA folding to short-range contacts, favouring the appearance of the PSs in HPeV1. These could then associate with their binding sites on a pentamer. As each protomer binds RNA, the genome would form a loop until the next PS sequence appeared. This could attach to a neighbouring binding site by chance, or pentamers could rotate relative to 2C to orientate the RNA at unbound sites. Once a pentamer’s RNA sites are full, a second pentamer may become associated forming the CP-CP contacts seen in the virion. Such a model accounts both for the data described here and previous models of picornavirus assembly

One way to alter folding propensity would be during replication, local secondary structures being more favoured as incomplete transcripts emerge from viral polymerases. In some picornaviruses, there is evidence that only nascent genomic transcripts are encapsidated^25^, whilst recognition of their 5′ IRES structure, marks them as mRNAs. Additionally, in poliovirus, the virally encoded 2C protein introduces nascent viral RNAs into the pentameric capsomers^2^. The PS sites identified here are entirely compatible with such systems. Our model (Fig. 6) is of an “assemblysome” that combines production of nascent genomes with RNA chaperoning by 2C, resulting in kinetic folding of the PS sites as they emerge from the replicase. The illustration in Fig. 6 depicts the essential molecular challenges the virus must overcome to assemble the virion.

Parechoviruses ideally lend themselves for PS identification via our SELEX-based bioinformatics approach. Many other picornaviruses undergo cleavage of VP0 that could dislodge the RNA-CP contacts seen in HPeV1. Although extensive genetic screening has been carried out in other picornavirus systems to look for RNA assembly determinants, the dispersed and sequence-degenerate nature of the PSs identified here, explain some of those negative results^1–3^. The selective advantages of the PS-mediated mechanism are manifold. They include being evolutionarily robust, and ensuring assembly fidelity and efficiency arising out of the collective action of multiple sites of varying affinity, rather than being dependent on a single site. If PSs are part of a co-operative assembly process, mutation of individual sites can be expected to have different levels of effect from zero to lethality depending on their neighbouring PSs as demonstrated by our transfection studies. In agreement with this, bacteriophage MS2, which was a paradigm of the classical single PS site, shows only a limited decrease in viral titre when that site is mutated^26^, rather than a non-assembling phenotype expected for a unique assembly initiation site. Multiple analyses have now shown that this is because of the presence of dispersed PSs assisting assembly^15,27,28^, and these PSs have been seen directly in a recent asymmetric EM reconstruction^29^. The fact that the HPeV1 CP interface contacting its multiple PSs is lined with amino acid side-chains that are completely conserved across the *Parechovirus* genus suggests that PSs with similar recognition motifs should occur across the entire genus. Comparable analysis across the known *Parechovirus* strains confirms this to be true.

In summary, the above analysis of the selected HPeV1 aptamer library to identify putative PSs in other sequence-conserved parechoviruses implies that the high degree of conservation of the protein target is mirrored by the conservation of the PS recognition motif in the *Parechovirus* genus, and potentially also in larger groups of picornaviruses. This suggests that the CP recognition motifs of the RNA-PSs would constitute good targets for novel broad-spectrum antivirals. A major challenge now is to determine whether the PS-mediated virion assembly mechanism, which appears a conserved function across the entire *Parechovirus* genus, extends even further in the *Picornaviridae* family: whether all picornaviruses assemble via a similar mechanism, or whether the *Parechovirus* genus is unique. It is possible that maturation of the polyprotein and other virion-wide conformational changes may mask these effects in viruses that cleave their polyprotein, as described above. In either case, our results open up the potential of an anti-viral therapy against a broad spectrum of picornaviruses, which has previously not been possible in any viral system at this scale. In addition, our new insights into the rules that govern the encapsidation of genomic RNA in this class of viruses may have utility in creating non-infectious VLPs, a crucial hurdle in vaccine design.

## Methods

### SELEX target and library preparation

Purified HPeV1 Harris strain was biotinylated in phosphate buffered saline (PBS), at a molar ratio of 1:20 of capsid lysines:biotin (NHS-LC-LC-biotin, Pierce), according to the manufacturer’s protocol. Pentamers were isolated by heating the biotinylated virus in 1× selection buffer (10 mM Tris-HCl pH 7.7, 150 mM NaCl and 1 mM MgCl_2_) at 56 °C for 30 min followed by incubation at 4 °C for 10 days. Whole capsids were removed by centrifugation (114,704×*g*, 10 min, RT). Biotinylated pentamers (180 µg) were immobilised on 300 µl 10 mg/ml streptavidin-coated paramagnetic beads (Dynabeads MyOne Streptavidin T1, Thermofisher Scientific) by rolling overnight at 4 °C. These beads (referred to as positive beads) were then washed with 1× selection buffer three times to remove excess protein. The beads were resuspended in 300 µl of 1× selection buffer to a final bead concentration of 10 mg/ml.

The naïve RNA library was transcribed from a synthetic combinatorial N40 (∼10^24^ potential sequences) double-stranded DNA library using HiScribe T7 High Yield RNA Synthesis kit (NEB). The DNA template was then removed by digestion with TURBO DNase (Thermofisher Scientific) and the RNA purified using RNAClean XP resin (Agencourt).

### SELEX protocol

Ten rounds of SELEX were performed using a Biomek2000 liquid-handling robot (Beckman Coulter). 50 µl of the purified RNA library (10 µM) was added to 50 µl 2× selection buffer and incubated with 20 µl of 10 mg/ml MyOne T1 Dynabeads with no immobilised protein for 15 min at 37 °C. The beads were partitioned via magnetic separation (2 min) and the unbound fraction removed and incubated with 20 µl target beads for 25 min at 37 °C. Beads were partitioned again (2 min) and the unbound fraction removed. Beads were washed ten times (1 min incubations) with 125 µl of selection buffer to remove unbound/weakly bound RNA species before being finally resuspended in 35 µl DEPC-treated H_2_O. 80 µl of mineral oil was dispensed on top of the mixture to avoid evaporation. Bound RNA was eluted from the beads by heating to 95 °C for 5 min in a PCR thermocycler. RNA was reverse transcribed to DNA using a Transcriptor Reverse Transcription kit (Roche). Samples were incubated at 52 °C for 1.5 h, followed by the addition of KAPA2G robust PCR mix (scaled up to 80 µl final volumes) and the enriched library PCR amplified for the next SELEX round. Stringency was increased across the ten rounds in three ways: 1. By decreasing the target bead incubation time over the first five rounds from 25 to 5 min; 2. For rounds 6–10, the amount of target beads was halved to 10 µl; 3. The number of washes was increased from 10 to 15 over rounds 6–10.

For rounds 5 and 10, a competition was carried out to remove RNA sequences that had a higher affinity for the capsid than for the inner pentamer surface. The SELEX round was performed as above but after the washes, the positive beads were incubated with either 0.1 mg/ml of biotinylated capsid (round 5) or capsid (round 10). A further three washes were carried out to remove the capsids and any capsid bound RNA aptamers. Elution, etc, was then performed as above. The amplified DNA of round 10 was then subjected to Next-Generation sequencing using Illumina MiSeq (Supplementary Fig. 1).

### Sequence alignment and computation of the Bernoulli score

Sliding the aptamer sequence along the genome in increments of 1 nt generated genome frames for comparison with the aptamer sequence. In order to treat information at the 5′ and 3′-end on an equal footing to the middle sections in the histogram plot, we also considered partial overlaps, obtained from alignments of the 3′ region of the aptamers to the 5′ regions of the genome and vice versa, gradually increasing in steps of 1 nt until full overlap was reached. For each such alignment, we calculated the maximum Bernoulli score, defined as *B(L,N)* = *L−*log_4_ *x* where ${x}=\sum _{i=0}^{N}(\mathop{L}\limits_{i})\ast {3}^{i}$ (ref. 30). This is related to the probability *P(L,N)* = *(1/4)* ^*B(L,N)*^ that a random sequence of *B(L,N)* letters would align precisely with the genome. Since typically the fragment contributing to the score was smaller than the length of the aptamer and contained some mismatches, we identified the largest fragment of the aptamer that had the highest Bernoulli score, and therefore, the lowest probability of having aligned to the genome fragment by chance. For each comparison frame, the fragment of the aptamer that aligned to the genome with the maximum Bernoulli score was identified via computation of the contributions of different mismatches to the scores of any contiguous subset of the frame. If this maximum score was larger or equal to 12, we logged it into the data file that was subsequently used to compute the histogram in Fig. 2a.

### Testing of mutated HPeV1 PS and capsid proteins

Mutations in the PSs and in the capsid protein regions were made in an infectious HPeV1 cDNA clone, pHPeV1 (kind gift of Petri Susi, University of Turku), using the Geneart site-directed mutagenesis system (Invitrogen) according to the manufacturer’s instructions^31^. The primers are listed in Supplementary Table 4.

Infectious genomic RNA was produced after transfection and translation in GMK cells. For this, the plasmids containing the cDNA clones of mutants or wild-type, were used as the templates to produce linear cDNA by PCR amplification using the T7 promoter-containing forward primer, 5′-TAATACGACTCACTATAGGGTTTGAAAGGGGTCTCCTAGAGAG-3′ and polyT-containing reverse primer 5′-TTTTTTTTTTTGTCATGTCCAATGTTCC-3′. The PCR running conditions were 98 °C (5 min), [98 °C (30 s) + 55 °C (30 s) + 72 °C (5 min)] × 25, 72 °C (10 min), 4 °C. The resulting linear cDNA contained the T7 promoter and the appropriate full-length HPeV1 cDNA genome.

To test these mutants in vivo, a plasmid containing the T7 RNA polymerase under the CMV promoter^32^ was transfected into GMK cells (kind gifts of Petri Susi) using Lipofectamine 2000 (Life Technologies, Cat# 11668027). Next day, these GMK cells were transfected with the linear cDNA, to generate full-length genomic RNA, which in the control experiments with the wild type cDNA, generates virions. Cell lysate was prepared from the transfected cells on the 3rd day post the second transfection by two cycles of freeze-thawing at −180 °C and 37 °C. The cell lysate was treated with RNase I (ThermoFisher Scientific, Cat# EN0601) and DNase I (ThermoFisher Scientific, Cat# EN0525) for 30 min at 37 °C to degrade any residual free RNA or DNA and subsequently added to HT29 cells (human colorectal adenocarcinoma cell line; Sigma, Cat# 91072201-1VL) and scored for CPE for 4 days. The viral titres were expressed as 50% tissue culture infectious dose (TCID50) per ml using an end-point titration method described earlier^33^. The HT29 cells are an indicator cell line which shows clear CPE for HPeV1 compared to GMK. The HT29 and GMK cells were tested for mycoplasma prior to use.

### RNA replication of the mutants

In order to gauge the efficiency of viral RNA and protein production in the GMK cells, the transfected cells were produced as for the above end point assay. In addition, cells were infected with HPeV1 virions (MOI 0.1) to serve as a positive control, and untransfected cells were used as a negative control.

In order to detect the formation of double-stranded viral RNA, an immunofluorescence assay was performed. The cells were fixed at 6 h post second transfection with 4% paraformaldehyde for 15 min at room temperature (RT). The cells were washed twice with 1× PBS and permeabilised with 0.1% Triton X-100 for 20 min at RT, followed by blocking (3% bovine serum albumin and 0.1% Tween-20 in 1× PBS) at RT for 1 h. The primary antibody incubation was done at 4 °C overnight with mouse J2 mAb against dsRNA (English and Scientific Consulting Kft., Cat# 10010200; stock: 1 mg/ml) at a 1:1500 dilution. The cells were washed three times with 1× PBS and incubated with a fluorescein isothiocyanate (FITC) labelled anti-mouse secondary antibody (Sigma) at 1:200 dilution. The cells were washed three times with 1× PBS. The 4′,6-diamidino-2-phenylindole (DAPI) stain (Sigma) at a working concentration of 0.1 µg/ml was added to the cells. The images were obtained with a Floid Cell Imaging Station (Life Technologies) in the Biocenter Finland Light Microscopy Unit, University of Helsinki.

On the 2nd day post the second transfection, the wild type, mutants, positive virus infection and negative controls from the GMK cells were used for both total RNA extraction with Trizol (Invitrogen) and the production of cell lysates by freeze-thawing.

The total RNA was used for cDNA synthesis with random hexamers utilising a Phusion RT kit (Thermo Scientific). The cDNA was subjected to quantitative real-time PCR (Roche) using primers and a probe designed for a 142bp-long conserved 5′ UTR region as described earlier^34^.

### In vitro translation of the mutants

Plasmids carrying the wild type genome (pHPeV1) or its mutants were transcribed and translated using the TNT Quick Couple Transcription/Translation system (Promega) as per the manufacturer’s protocol. A plasmid carrying the luciferase gene (provided in the kit) was used as the positive control. The proteins were labelled with ^35^S-methionine. The signal was obtained on a phosphoimager after overnight exposure.

### Microscale thermophoresis (MST)

We generated 5′-Alexa-488-labelled RNA oligomers based on the genome sequences for the PSs (PS6, PS7, PS9, PS14 and PS21) and their sequence variants and measured their affinities for HPeV1 pentamers, ranging in concentration from ~0.5–900 nM pentamer, using a microscale thermophoresis Monolith machine (NanoTemper)^35^. Each pentamer dilution was mixed with an equal volume of 50 nM of RNA (final concentration 25 nM), incubated for 1 h at 37 °C, and subjected to thermophoresis using the standard, untreated, capillaries. Conditions for MST: 5 s LED on (Blue LED), 35 s laser on, 5 s recovery. A control was also performed using the MS2 CP and its highest affinity PS, the translational operator, TR RNA. The MST signal is a composite of positive and negative thermophoretic effects that are acutely sensitive to the nature and solution environment of the dye-labelled species. If multiple self-association occurs, as was observed here, the signal can reverse preventing accurate *K*_d_ determination. The binding data were therefore assessed semi-quantitatively (+/−) noting the amplitude of signal change over the range of pentamer concentrations and where significant binding began to occur. These were compared to a known PS-CP interaction, that of bacteriophage MS2 TR and its CP (*K*_d_ ~1–4 nM, depending on binding assay used), which was designated as +++++. The highest protein concentration samples from these assays were recovered, negatively-stained and examined by electron microscopy.

### Crystal structure model building

The coordinates and structure factors for the X-ray crystal structure of human parechovirus were obtained from the PDB (accession code 4Z92)^4^. The un-averaged 2mF_o_−DF_c_ map was of sufficient quality for structural interpretation, without further real-space averaging being required. Close inspection of the deposited model revealed that portions of the RNA molecule and some of the surrounding amino acid side-chains were not optimally modelled into the electron density. These errors were corrected by manual rebuilding in COOT^36^ and 10 cycles of refinement in REFMAC^37^ with the X-ray weight set to 0.008 and strict ten-fold non-crystallographic symmetry applied. Structures were refined in parallel with either A or G in position 1 of the RNA molecule, to *R*_work_/*R*_free_ values of 0.258/0.261 and 0.259/0.262 respectively, and root-mean-square deviations in bond lengths/angles of 0.007 Å/1.25° (see Supplementary Table 5 for refinement statistics and Supplementary Fig. 7 for stereo-view of the refined model in the density). The refined structures were validated using the MOLPROBITY server^38^. For the model with G in position 1 of the RNA molecule, the MOLPROBITY score was 1.86 (100% percentile), with 93.8%/5.8%/0.4% of residues lying in favoured/allowed/forbidden regions of the Ramachandran plot. For the model with A in position 1 of the RNA molecule, the MOLPROBITY score was 1.86 (100% percentile), with 93.7%/5.9%/0.4% of residues lying in favoured/allowed/forbidden regions of the Ramachandran plot.

### Code availability

Computer code for sorting the next generation sequencing data and producing the sequence alignment plots is available upon request.

### Data availability

The refined model for HPeV1 has been deposited in the Protein Data Bank in Europe under accession code 5MJV. The authors declare that all other data are available from the authors upon request.

## Electronic Supplementary Material

Supplementary Information Supplementary Figures, Supplementary Tables and Supplementary References

Peer Review File Reviewer reports and authors' response from the peer review of this Article at Nature Communications
