# Erratum: Genomic RNA folding mediates assembly of human parechovirus

**DOI:** 10.1038/s41467-017-00026-4

**Published:** 2017-07-14

**Authors:** Shabih Shakeel, Eric C. Dykeman, Simon J. White, Ari Ora, Joseph J. B. Cockburn, Sarah J. Butcher, Peter G. Stockley, Reidun Twarock

**Affiliations:** 1 Institute of Biotechnology and Department of Biosciences, University of Helsinki, Helsinki, 00790 Finland; 2 Departments of Mathematics and Biology and York Centre for Complex Systems Analysis, University of York, York, YO10 5DD UK; 3 Astbury Centre for Structural Molecular Biology, University of Leeds, Leeds, LS2 9JT UK; 4 Department of Applied Physics and Department of Biotechnology and Chemical Technology, Aalto University, Espoo, 02150 Finland

*Nature Communications* **8**:5 doi: 10.1038/s41467-016-0011-z; Article published online 23 Feb 2017

The HTML version of this paper was updated shortly after publication, following an error that resulted in incorrect versions of Figs 1 and 2 being displayed. The figures have now been corrected in the HTML; the PDF version of the paper was correct from the time of publication.

