# Supplementary material for: Genomic RNA folding mediates assembly of human parechovirus
Source: Nat Commun. 2017 Feb 23;8:5. doi: 10.1038/s41467-016-0011-z (PMC5431903; doi:10.1038/s41467-016-0011-z)
Supplement: Supplementary file 1 — Supplementary Figures, Supplementary Tables and Supplementary References [file 41467_2016_11_MOESM1_ESM.pdf]

Supplementary figures

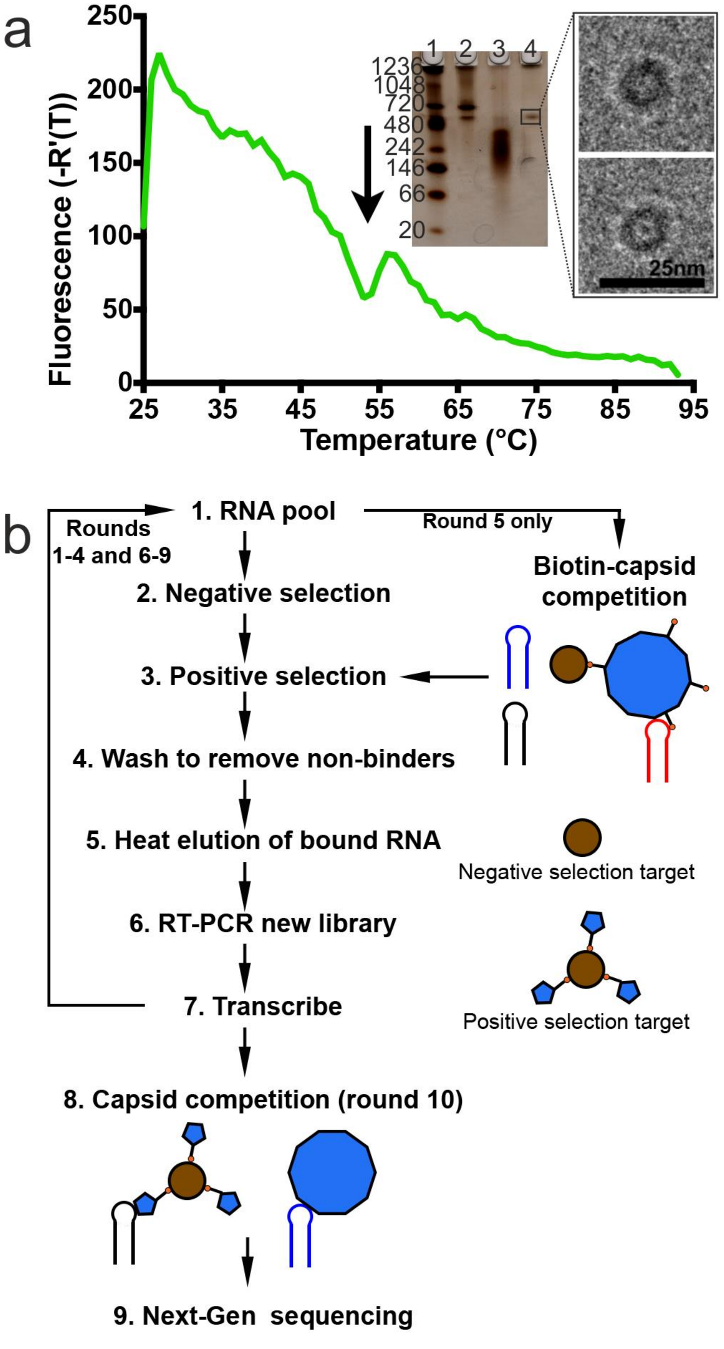

Supplementary Figure 1. SELEX protocol.

(a) A thermofluor assay was used to identify the temperature at which the capsid destabilizes using Sybr Safe DNA dye (Invitrogen) which fluoresces upon binding to the RNA[1]. The arrow indicates the temperature at which RNA becomes accessible to the dye. Contrary to expectation, heating at 56°C did not result in empty capsids, but instead, resulted in a smaller intermediate of ~431 kDa in

size. The size was detected on a silver-stained, native, 4-20% precast polyacrylamide gel shown as an inset. Lane 1, NativeMark Unstained Protein Standard cat# LC0725 from Life technologies with the molecular weights in kDa marked on the gel; lane 2, thyroglobulin, molecular weight 669 kDa; lane 3, β-amylase, molecular weight 200 kDa; HPeV1 heated at 56°C for 30 minutes. When the sample was examined in cryoEM[1], it resembled a pentamer (micrograph inset). (b) Biotinylated pentamers (blue pentagons) were immobilized on streptavidin magnetic beads (brown circles) and RNA aptamers (coloured hoops) selected by repeated cycles of binding, partition and amplification. Negative selections at each round used streptavidin Dynabeads coated with Tris-inactivated linker. Stringency was increased after round 5 by decreasing the number of pentamer-coated Dynabeads by half and increasing the number of washes in rounds 6 to 10 by one wash each round, i.e. by round 10 there were 15 washes. At the end of round 5, a counter selection was performed against biotinylated whole capsid (blue decagon) whereby the selected RNA library (50µl) was mixed with the capsid (50 µl at 0.1 mg/ml) for 5 min at 37°C, and the biotinylated capsid and any associated RNA were then captured on streptavidin Dynabeads. The unbound RNA was then used in a normal round of SELEX. The final round (round 10) was performed as normal but before heat elution of the RNA library from the positive beads, the positive beads were challenged with 100 µL of whole capsid (0.1 mg/mL) for 5 minutes at 37°C. After three washes to remove any remaining capsid/RNA complexes the positive beads were heat eluted as normal. The reverse transcriptase-PCR products, taken at the end of each round, were analyzed by native PAGE to confirm the isolation of products for the next round of selection.

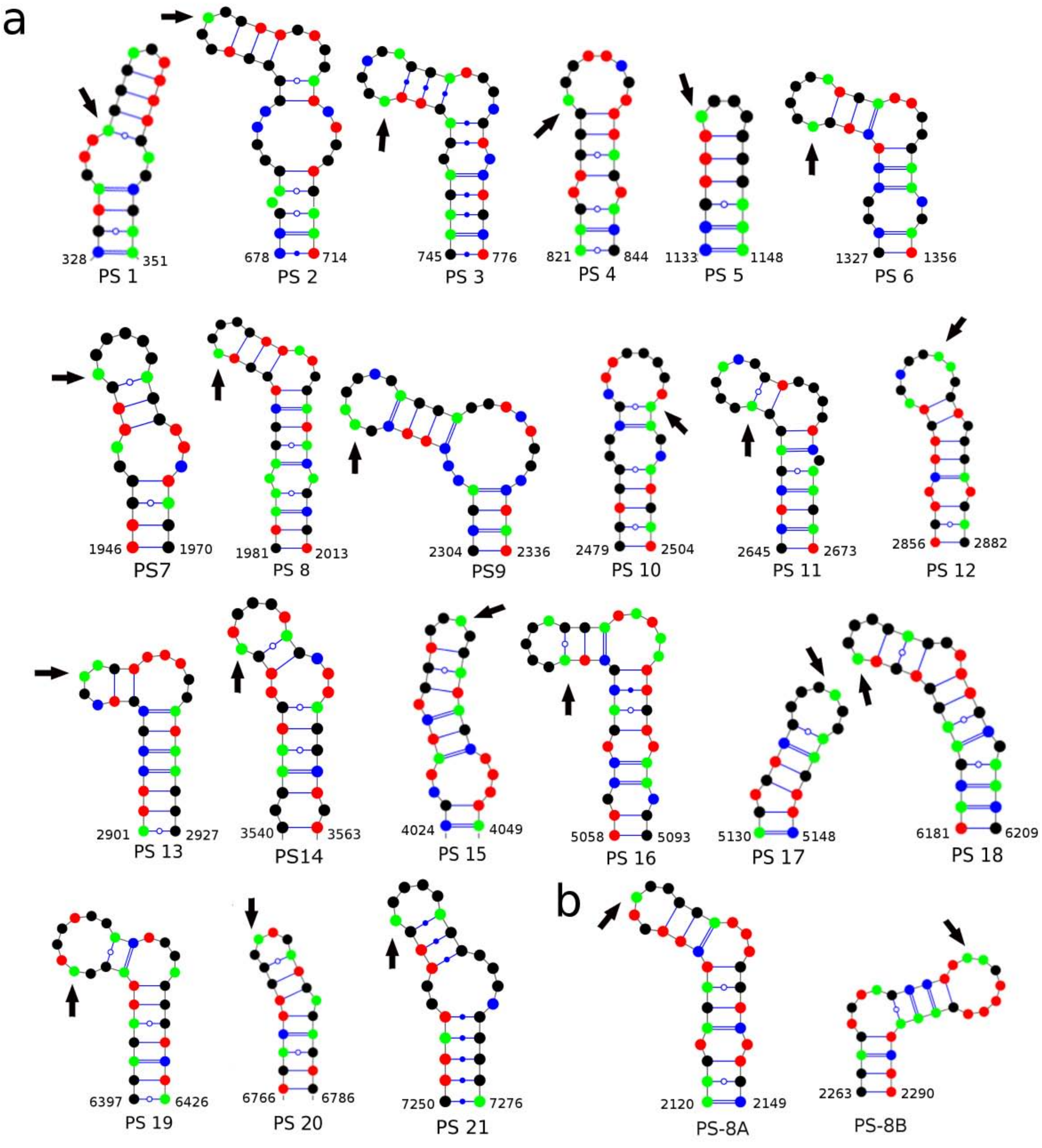

Supplementary Figure 2. **Putative Packaging Signals corresponding to the Bernoulli Peaks in the Harris strain.**

(a) Fragments plus/minus 20 nucleotides around the 21 Bernoulli peaks in Fig. 2a can fold into stem-loops containing the GxU motif in the loop portion, consistent with the HPeV1 protein:RNA

interactions in Fig. 3b. Only the predicted Mfold secondary structures are shown for each PS, with numbers indicating starting and ending nucleotides in the Harris strain. Bernoulli peak positions of PS1 to PS21 are (in nucleotides): 338, 693, 753, 827, 1142, 1344, 1957, 1995, 2322, 2493, 2660, 2873, 2919, 3553, 4037, 5073, 5139, 6194, 6409, 6778, 7264. (b) The two peaks below the cut-off defined by the naïve library, marked by asterisks in Fig. 2a, can also fold into stem-loops with a GxU motif in the loop portion, suggesting that peaks below the cut-off could also be relevant, even though this cannot be assumed a priori. There are 56 such peaks in total, which is close to the 60 expected from the RNA density distribution in the virion. The arrows in (a) and (b) indicate the start of the GxU motifs within the single-stranded regions of the PS loops. There are three exceptions, PS11 and PS16 where the G is the base-pair 5´ to the start of the loop and PS10 where the G is base-paired 3´ to the central loop. The latter is interesting because it has no matches within any other strain variant (see Fig. 2b). Mfold structures are drawn in VARNA[2], with G – green; C – blue; A – red and U – black.

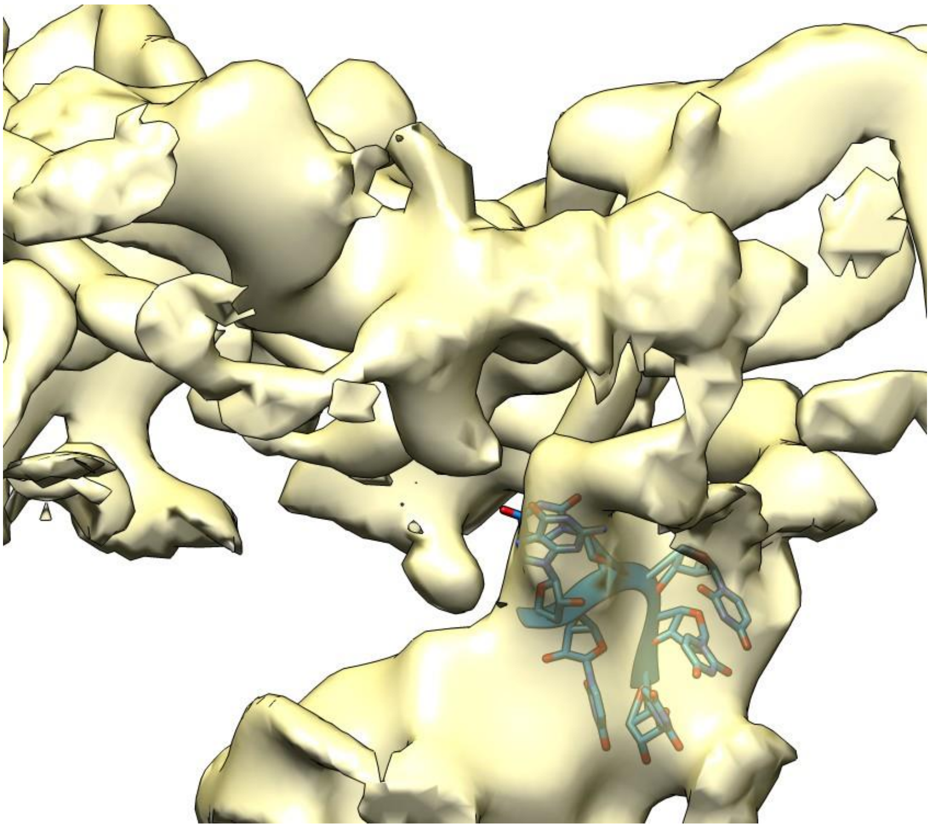

Supplementary Figure 3. RNA model in the HPeV1 EM density.
A close-up view showing a ribbon and all-atom model of the ordered RNA hexanucleotide from Figure 3b superimposed into the EM density of HPeV1 (EMDB ID: 1690, yellow transparent isosurface representation)[3]. The ordered EM density below this fragment (density at the bottom of the figure) would not be seen if the sequences flanking the GxU recognition motif were not capable of forming a base-paired stem. An A-duplex model of such a stem fits well into the EM map (not shown), consistent with the equivalent sites in HPeV3[4].

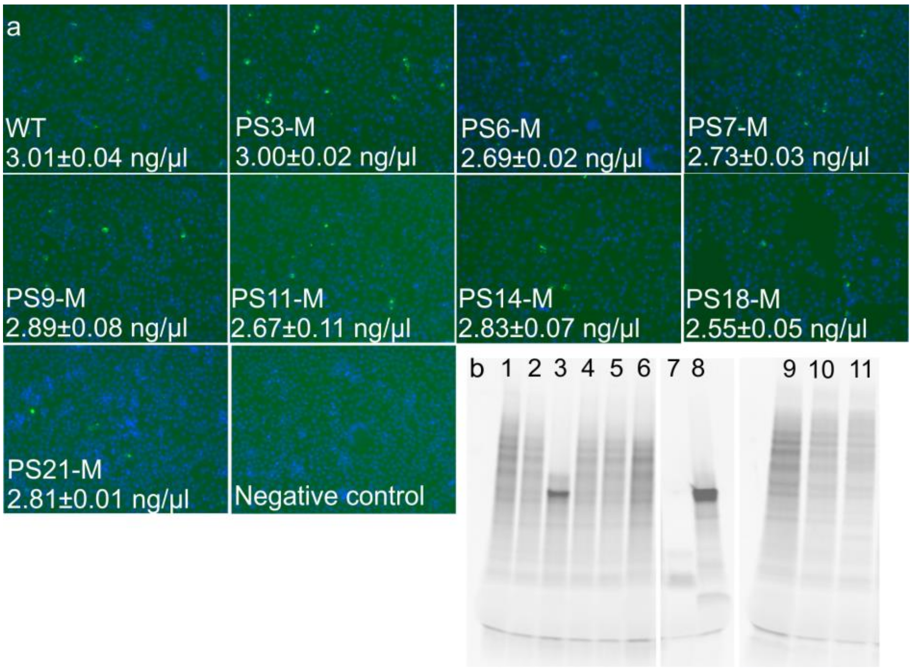

Supplementary Figure 4. Replication and translation of PS mutants.
(a) Immunofluorescence assay with anti-dsRNA antibody to detect virus replication foci. The assay was performed 6 h post-transfection with clones of wild-type (WT) and the mutants, all of which were positive (green; DAPI staining, blue). The average viral RNA concentration (mean ± standard deviation) as determined by quantitative real time PCR is marked in each panel. (b) In vitro translation from the WT and mutant clones. The lanes are marked as follow: 1, WT; 2, PS3-M; 3, PS9-M; 4, PS11-M; 5, PS18-M; 6, PS21-M; 7, negative control; 8, positive control for the *in vitro* translation assay; 9, PS6-M; 10, PS7-M; 11, PS14-M.

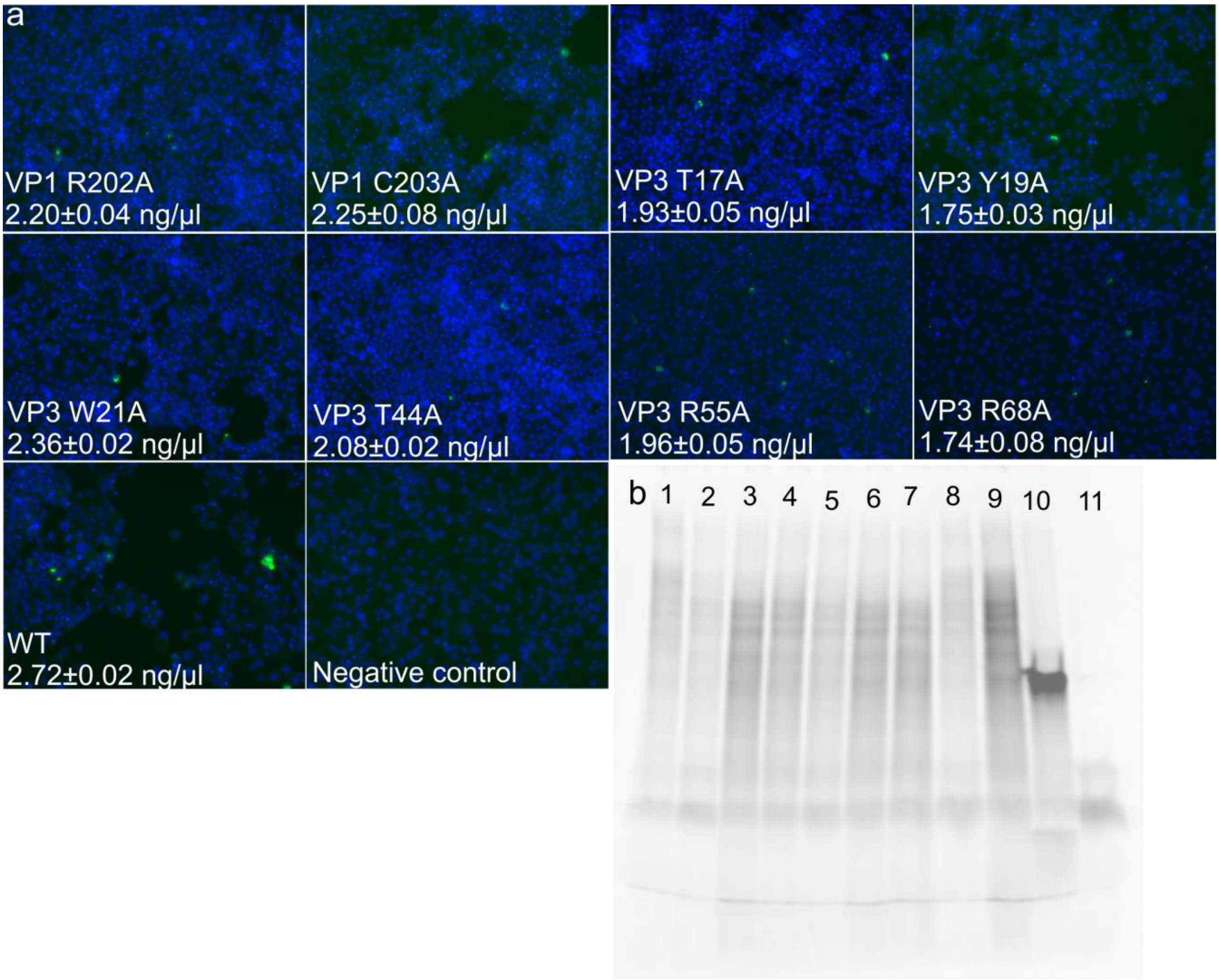

Supplementary Figure 5. Replication and translation of VP3 and VP1 mutants.

(a) Immunofluorescence assay with anti-dsRNA antibody to detect virus replication foci. The assay was performed 6 h post-transfection with clones of wild-type (WT) and the mutants, all of which were positive (green; DAPI staining, blue). The average viral RNA concentration (mean ± standard deviation) as determined by quantitative real time PCR is marked in each panel. (b) In vitro translation from the WT and mutant clones. The lanes are marked as follow: 1, VP3 T17A; 2, VP3 Y19A; 3, VP3 W21A; 4, VP3 T44A; 5, VP3 R55A; 6, VP3 R68A; 7, VP1 R202A; 8, VP1 C203A; 9, WT; 10, positive control for the *in vitro* translation assay; 11, negative control.

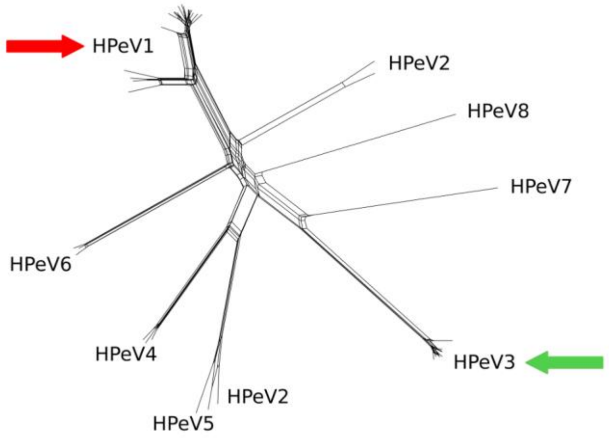

Supplementary Figure 6. Phylogenetic tree based on the structural protein sequences of human parechovirus genomes from groups 1 to 8.

A phylogenetic tree based on the VP1-VP4 coding sequences is shown for full human parechovirus genome sequences available from GenBank (see Supplementary Table 3), created using SplitsTree. Even though the structural proteins in the Harris strain in the HPeV1 group (red arrow) are most distal from those in the HPeV3 group (green arrow), a representative viral genome in the HPeV3 group is still showing excellent matches with the selected HPeV1 library and PS recognition motif.

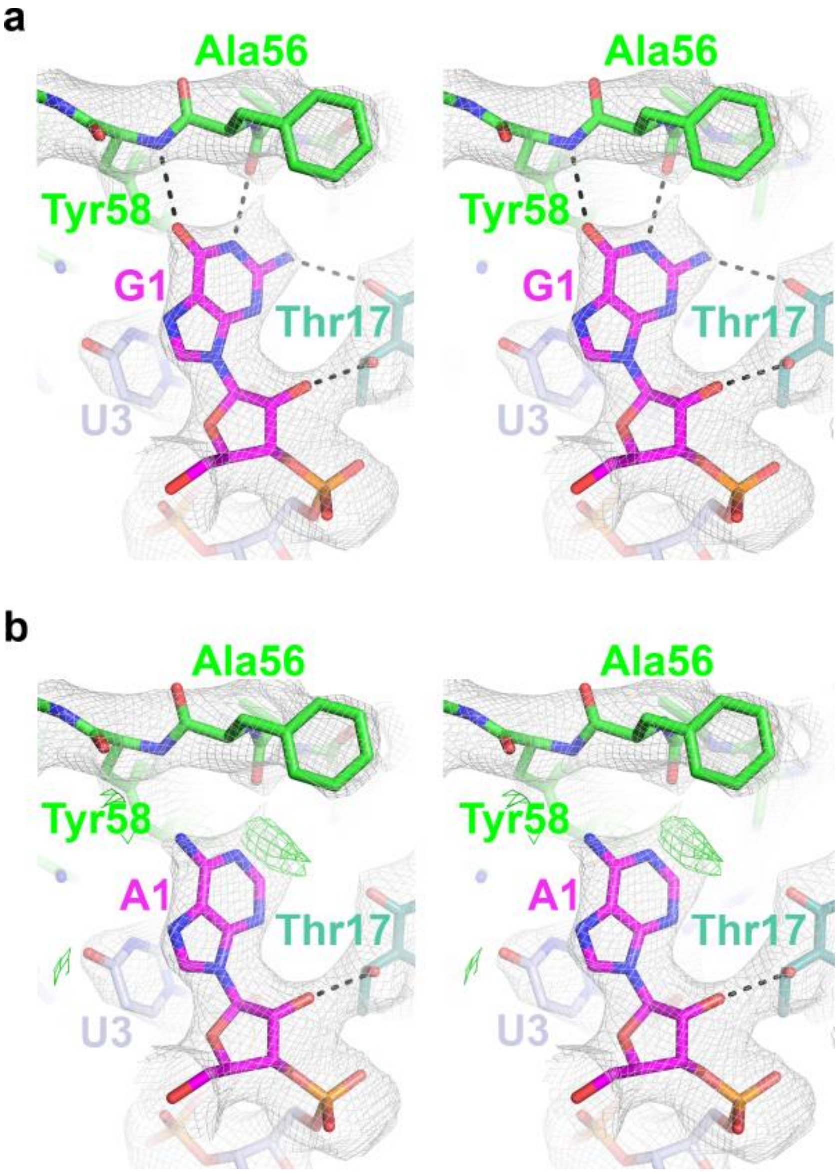

Supplementary Figure 7: Stereo-views of Fig 3a.

Stereo views of the refined atomic models of the virus with guanosine (a), and adenosine (b) modelled in position 1 of the RNA.

Supplementary Tables.

Supplementary Table 1. Accession codes for sequences of HPeV1 strains used in genome analysis

**Strain number[a]**	**Genbank ID**
Harris strain	L02971.1
Strain 2	JX441355.1
Strain 3	JX575746.1
Strain 4	EF051629.2
Strain 5	FJ840477.1
Strain 6	GQ183035.1
Strain 7	GQ183025.1
Strain 8	GQ183023.1
Strain 9	GQ183021.1
Strain 10	GQ183019.1
Strain 11	GQ183034.1
Strain 12	GQ183024.1
Strain 13	GQ183022.1
Strain 14	GQ183020.1
Strain 15	GQ183018.1
Strain 16	HQ696574.1
Strain 17	HQ696572.1
Strain 18	HQ696570.1
Strain 19	HQ696573.1
Strain 20	HQ696571.1
Strain 21	FM178558.1

[a]The sequences of strains 1-21 referred to in Fig. 2b were retrieved from Genbank with the following Genbank IDs.

Supplementary Table 2. Van de Waals interactions between RNA and capsid

RNA			Protein				
Base	**Identity**	**Atom**	**Subunit**	**Residue**	**Identity**	**Atom**	**Distance**
1	G	O2'	VP3[c]	17	THR	CB	3.64
			VP3[c]	17	THR	CG2	3.96
1	G	N3	VP3	55	ARG	NH2	3.77
1	G	C2	VP3	56	ALA	O	3.74
1	G	N2	VP3[c]	17	THR	O	3.31
			VP3	55	ARG	CB	3.86
			VP3	56	ALA	O	3.38
			VP3	55	ARG	NE	3.65
			VP3	55	ARG	CZ	3.95
			VP3	55	ARG	NH2	3.75
1	G	N1	VP3	58	TYR	CD2	3.93
1	G	C6	VP3	58	TYR	CD2	3.72
1	G	O6	VP3	57	PHE	CA	3.71
			VP3	57	PHE	C	3.82
			VP3	58	TYR	CD2	3.42
			VP3	58	TYR	CA	3.93
			VP3	58	TYR	CB	3.83
			VP3	58	TYR	CG	3.91
2	U	OP2	VP3[c]	17	THR	CB	3.9
			VP3[c]	17	THR	OG1	3.61
3	U	C4'	VP1	203	CYS	SG	3.78
3	U	O3'	VP3	55	ARG	NH2	3.71
			VP1	203	CYS	SG	3.88
3	U	C2'	VP3	55	ARG	NH2	3.6
3	U	O2'	VP3[c]	17	THR	CG2	3.95
			VP3	55	ARG	CZ	3.81

3	U	C1'	VP3	55	ARG	NH2	3.6
3	U	O4'	VP3	68	ARG	NH2	3.4
			VP1	203	CYS	SG	3.47
3	U	N1	VP3	68	ARG	NH2	3.85
3	U	C6	VP3	68	ARG	NH2	3.58
3	U	C5	VP3	68	ARG	NE	3.8
			VP3	68	ARG	NH2	3.89
3	U	C4	VP1	205	ASN	ND2	3.74
			VP3	68	ARG	NE	3.76
3	U	N3	VP1	205	ASN	CG	3.89
			VP1	205	ASN	OD1	3.49
			VP1	205	ASN	ND2	3.47
3	U	O2	VP3	58	TYR	CE2	3.58
			VP1	205	ASN	OD1	3.85
			VP1	203	CYS	CB	3.87
			VP3	55	ARG	NH2	3.43
3	U	O4	VP3	68	ARG	CG	3.82
4	U	P	VP1	202	ARG	NH2	3.9
4	U	OP1	$VP3^{c}$	17	THR	CG2	3.74
4	U	C5'	VP1	202	ARG	NH2	3.84
			VP1	202	ARG	NH1	3.63
4	U	O2'	VP3	41	LEU	CD2	3.49
4	U	C1'	$VP3^{c}$	21	TRP	CD1	3.59
			$VP3^{c}$	21	TRP	NE1	3.97
4	U	O4'	$VP3^{c}$	21	TRP	CG	3.96
			$VP3^{c}$	21	TRP	CD1	3.67
4	U	N1	$VP3^{c}$	21	TRP	CG	3.75
			$VP3^{c}$	21	TRP	CD1	3.49
			$VP3^{c}$	21	TRP	NE1	3.59
			$VP3^{c}$	21	TRP	CE2	3.9
			$VP3^{c}$	21	TRP	CD2	3.99

4	U	C6	$VP3^{c}$	21	TRP	CD1	3.79
			$VP3^{c}$	21	TRP	NE1	3.36
			$VP3^{c}$	21	TRP	CE2	3.55
			$VP3^{c}$	21	TRP	CZ2	3.99
4	U	C5	$VP3^{c}$	21	TRP	NE1	3.78
			$VP3^{c}$	21	TRP	CE2	3.48
			$VP3^{c}$	21	TRP	CZ2	3.5
4	U	C4	$VP3^{c}$	21	TRP	CE2	3.79
			$VP3^{c}$	21	TRP	CD2	3.86
			$VP3^{c}$	21	TRP	CE3	3.95
			$VP3^{c}$	21	TRP	CZ3	3.95
			$VP3^{c}$	21	TRP	CH2	3.89
			$VP3^{c}$	21	TRP	CZ2	3.82
4	U	N3	$VP3^{c}$	21	TRP	CD2	3.75
			$VP3^{c}$	21	TRP	CE3	3.9
4	U	C2	$VP3^{c}$	21	TRP	CG	3.69
			$VP3^{c}$	21	TRP	CD1	3.93
			$VP3^{c}$	21	TRP	CD2	3.86
4	U	O2	$VP3^{c}$	21	TRP	CB	3.85
			$VP3^{c}$	21	TRP	CG	3.92
4	U	O4	$VP3^{c}$	21	TRP	CZ3	3.76
			$VP3^{c}$	21	TRP	CH2	3.76
4	U	OP2	VP1	202	ARG	CZ	3.68
5	U	C4'	$VP3^{b}$	19	TYR	OH	3.91
5	U	O3'	$VP3^{b}$	19	TYR	CZ	3.58
			$VP3^{b}$	19	TYR	CE2	3.48
5	U	O2'	$VP3^{b}$	19	TYR	CE2	3.9
6	U	P	$VP3^{b}$	19	TYR	OH	3.78
6	U	OP1	$VP3^{b}$	19	TYR	CZ	3.99
			$VP3^{b}$	19	TYR	CE2	3.77
6	U	C5'	$VP3^{b}$	18	LYS	CE	3.91

Supplementary Table 3. Accession codes and group affiliations for HPeV genotypes used in the bioinformatics analysis

**Group 1**	Seq1: L02971.1; Seq2: JX441355.1; Seq3: JX575746.1; Seq4: EF051629.2; Seq5: FJ840477.1; Seq7: GQ183025.1; Seq8: GQ183023.1; Seq9: GQ183021.1; Seq10: GQ183019.1; Seq11: GQ183034.1; Seq12: GQ183024.1; Seq13: GQ183022.1; Seq14: GQ183020.1; Seq15: GQ183018.1; Seq16: HQ696574.1; Seq17: HQ696572.1; Seq18: HQ696570.1; Seq19: HQ696573.1; Seq20: HQ696571.1
**Group 2**	Seq22: HM996978.1; Seq23: AF055846.1; Seq24: AJ005695.1
**Group 3**	Seq25: KM986843.1; Seq26: KJ659490.1; Seq27: JX826607.1; Seq28: JX682576.1; Seq29: GQ183033.1; Seq30: GQ183032.1; Seq31: GQ183031.1; Seq32: GQ183030.1; Seq33: GQ183027.1; Seq34: AB084913.1; Seq35: AJ889918.1
**Group 4**	Seq36: AB433629.1; Seq37: DQ315670.1; Seq38: AM235750.1
**Group 5**	Seq39: JX050181.1; Seq40: HQ696575.1
**Group 6**	Seq41: FJ888592.1; Seq42: AB252582.1; Seq43: EU077518.1
**Group 7**	Seq44: EU556224.1
**Group 8**	Seq45: EU716175.1

Supplementary Table 4. Primers used for site-directed mutagenesis in the infectious cDNA clone of HPeV1

**Primer name**	**Forward primer (5´ to 3´)**	**Reverse primer (5´ to 3´)**
PS3-M	TTGCAGATATGGCTACTGGTGTTGTCAGCAGCGTTGATAGCACCATCAATGCAGTCAATGAAAAGG	CCTTTTCATTGACTGCATTGATGGTGCTATCAACGCTGCTGACAACACCAGTAGCCATATCTGCAA
PS6-M, set 1	TCACTTATGATTCAAAACTGGAATTCGGAGCTTTTACGAACCTTCCACATGTTTTGATGAATTTGG	CCAAATTCATCAAAACATGTGGAAGGTTCGTAAAAGCTCCGAATTCCAGTTTTGAATCATAAGTGA
PS6-M, set 2	AACCTTCCACATGTTCTTATGAACCTGGCTGAAACCACAC	GTGTGGTTTCAGCCAGGTTCATAAGAACATGTGGAAGGTT
PS7-M, set 1	TATTGTCCACAGAAATATTGTCTACCTGAGACTGTTCCCAAACTTGAATGTTTTTGTTAACAGTTATT	AATAACTGTTAACAAAAACATTCAAGTTTGGGAACAGTCTCAGGTAGACAATATTTCTGTGGACAATA
PS7-M, set 2	TGAGACTGTTCCCAAACTTGAATGTGTTTGTGAACAGTTACAGTTACTTTAGGGGTTCATTAGTTTTAA	TTAAAACTAATGAACCCCTAAAGTAACTGTAACTGTTCACAAACACATTCAAGTTTGGGAACAGTCTCA

PS9-M	CTTCTGCCCAACTGGAAGTGTAGTAACATTCCAGAATTCA	TGAATTCTGGAATGTTACTACACTTCCAGTTGGGCAGAAG
PS11-M	AACAAGGTCATGGGTCCTTATCACTACTCTTCGCCTACTTCACTGGTGAACTGAATATCCATGTTC	GAACATGGATATTCAGTTCACCAGTGAAGTAGGCGAAGAGTAGTGATAAGGACCCATGACCTTGTT
PS14-M, set 1	AATGTACTACATTCTTTTCTTCTGCCGCGACCGAGATTCTTGATAACGATTTAGTCAAGTTCATAGTGAAAA	TTTTCACTATGAACTTGACTAAATCGTTATCAAGAATCTCGGTCGCGGCAGAAGAAAAGAATGTAGTACATT
PS14-M, set 2	ATTCTTGATAACGATCTCGTCAAGTTCATAGTG	CACTATGAACTTGACGAGATCGTTATCAAGAAT
PS18-M	ATTCTATGGATTTGAGCACTTCAGCCGGATACTCCTTCGTCAAAAGCGGCTACAAAAAGAAAGATCTAATTT	AAATTAGATCTTTCTTTTTGTAGCCGCTTTTGACGAAGGAGTATCCGGCTGAAGTGCTCAAATCCATAGAAT
PS21-M	TGATGCCCATGGTGTTTGATTAAGACGAATGAAACGTTCGTCTTTTGCTATGGACTATGTGGTTA	TAACCACATAGTCCATAGCAAAAGACGAACGTTTCATTCGTCTTAATCAAACACCATGGGCATCA
VP1 R202A	GTTTATCTTAGCCTGGCATGTCCAAATTTCTT	AAGAAATTTGGACATGCCAGGCTAAGATAAAC
VP1 C203A	TATCTTAGCCTGAGAGCTCCAAATTTCTTTTT	AAAAAGAAATTTGGAGCTCTCAGGCTAAGATA
VP3 T17A	ATTATGACCATGAGCGCAAAATACAAATGGA	TCCATTTGTATTTTGCGCTCATGGTCATAAT
VP3 Y19A	ACCATGAGCACAAAAGCCAAATGGACCAGAAC	GTTCTGGTCCATTTGGCTTTTGTGCTCATGGT
VP3 W21A	AGCACAAAATACAAAGCGACCAGAACAAAAAT	ATTTTTGTTCTGGTCGCTTTGTATTTTGTGCT
VP3 T44A	AATGTCCTTTGTACAGCTGGTGCTCAATCAG	CTGATTGAGCACCAGCTGTACAAAGGACATT
VP3 R55A	GCTTTAGTAGGTGAAGCAGCTTTCTATGATCC	GGATCATAGAAAGCTGCTTCACCTACTAAAGC

VP3<br>R68A	GCAGGCAGTAAATCAGCATTTGATGATC<br>TTGT	ACAAGATCATCAAATGCTGATTTACTGCCTG<br>C

Supplementary Table 5. Refinement statistics

**Refinement**	G1	A1
R_{work} / R_{free}	0.258/0.261	0.259/0.262
No. atoms		
Protein/RNA	5365/120	5365/119
Ligand/ion	0	0
Water	0	0
B-factors (Å^2)		
Protein/RNA	46.2/61.4	46.5/61.6
Ligand/ion	-	-
Water	-	-
R.m.s deviations		
Bond lengths (Å)	0.0071	0.0071
Bond angles (°)	1.2522	1.2531

The collection statistics were published by Kalynych *et al*. 2015[5].

Supplementary References

1 Shakeel, S. *et al.* Structural basis of human parechovirus neutralization by human monoclonal antibodies. *J Virol* **89**, 9571-9580, doi:10.1128/JVI.01429-15 (2015).

2 Darty, K., Denise, A. & Ponty, Y. VARNA: Interactive drawing and editing of the RNA secondary structure. *Bioinformatics* **25**, 1974-1975, doi:10.1093/bioinformatics/btp250 (2009).

3 Seitsonen, J. *et al.* Interaction of alphaVbeta3 and alphaVbeta6 integrins with human parechovirus 1. *J Virol* **84**, 8509-8519, doi:10.1128/JVI.02176-09 (2010).

4 Shakeel, S. *et al.* Multiple capsid-stabilizing interactions revealed in a high-resolution structure of an emerging picornavirus causing neonatal sepsis. *Nat Commun* **7**, 11387, doi:10.1038/ncomms11387 (2016).

5 Kalynych, S., Palkova, L. & Plevka, P. The structure of Human Parechovirus-1 reveals an association of the RNA genome with the capsid. *J Virol*, doi:10.1128/JVI.02346-15 (2015).
