# Supplementary material for: Genomic RNA folding mediates assembly of human parechovirus
Source: Nat Commun. 2017 Feb 23;8:5. doi: 10.1038/s41467-016-0011-z (PMC5431903; doi:10.1038/s41467-016-0011-z)
Supplement: Supplementary file 2 — Reviewer reports and authors' response from the peer review of this Article at Nature Communications [file 41467_2016_11_MOESM2_ESM.pdf]

**<u>Reviewers' comments:</u>**

**Reviewer #1 (Remarks to the Author):**

A low resolution cryoEM structure for HPeV1 (8 angs) was supplemented recently with a higher resolution (3.1 angs) X-ray structure, allowing the Aus to create a composite model for the RNA binding sites inside the capsid, visualized as density in the cryoEM structure. This ms describes how that model guided SELEX selection and other experiments on genome sequence aptamers in an attempt to identify relevant RNA packaging signals. The best identified units contain a short consensus motif, GxU, as is consistent with observed densities, structure modeling and sequence variation within this genus of picornaviruses. In total, this is a novel approach, with results likely to influence our understanding of generic assembly processes for RNA viruses. It remains to be seen how far the findings can be extrapolated beyond this particular species of parechoviruses, because other picornaviruses generally undergo extensive internal protein rearrangements and RNA-catalyzed cleavages which surely influence their assembly processes, relative to the simple model proposed here.

Points which might strengthen the paper.

1. the SELEX amplification is not particularly impressive ("~400,000 were unique"). Since each of the protomer's protein sequence/structures is identical, isn't this telling you their individual selection of RNA sequences is actually quite promiscuous and/or involves only very small segments? One might actually interpret Fig 2 as a validation of promiscuity rather than specificity.

2. The Aus assume the interiors of pentamers from disassembled virions (used in the SELEX assays) are identical with regard to assembly competence to native pentamer precursors which have never seen packaged RNA. Is it possible, because of induced fit (see below), these (essentially) pre-trained pentamers might have more restrictive RNA binding references than naive pentamers? Most certainly this would be true for any picornavirus which undergoes VP0 cleavage. Although beyond the scope of the current ms, they may wish to comment on whether baculo-produced or in vitro-produced pentamers might necessarily select the same aptamers. (any pico protomer will make pentamers if properly cleaved by 3C.) That's the essential question here, isn't it? All the discussions about conserved (or not) structure motifs is pretty much hand-waving if the fit primarily relies on induction, nucleated around almost any GxU.

3. Extensive rearrangement of VP1, VP0 and VP3 protein tails in presence/absence of RNA (i.e. between full/empty particles) is well documented for multiple picornaviruses in both X-ray or cryo-EM structures, regardless of whether the resolved units were self-assembling pre-particles (i.e. never contained RNA), or had been induced to release their RNA (e.g. A-particles). Therefore, the static environment imaged for the interior proteins (current structure) in any full particles (or pentamers) is sure to be quite different than that encountered by the RNA during the assembly process itself. The Fig5 cartoon pretty much ignores the possibility that the RNA/protein fit is mostly likely highly induced, protomer-by-

protomer, or RNA motif-by-motif. Were this not the case, there should be 60 nearly equivalent (sequence and 2D structure) RNA motifs, waiting to be strung, unit by unit, into the next available protomer slot. But the observation is, some aptamers are better than others which means the process has to rely on a merely tolerable recognition with the next sequential GxU-like sequence unit deemed capable of induced fit with the next pentamer/protomer. The Aus should acknowledge in Results/Discussion (of their model) that extensive, progressive protein/RNA co-rearrangements are likely to strongly influence the overall assembly process. Their extensive search for commonalities in 2D RNA motifs really becomes moot if this is the case.

4. The EM on which key RNA assumptions are based is only at 8.5 angs. This fuzzy density and/or expected G/A first-base were modeled into the 3.1 angs X-ray protein map which itself has poorly resolved RNA densities. Ideally, one would do this exercise using a single much better resolution cryoEM dataset (currently possible at <3.0 angs) instead of superimposed amalgamated manual rebuild. Therefore, the juxtaposition here, while creative, seems to rely on a good deal of unsubstantiated, subjective modeling. Were these results accepted because they fit the Aus expectations?

5. Were the putative RNA 2D motifs returned by mFOLD, as shown in Fig2, Fig6 and S2 all done on local segments (60 b?) of the RNA sequence, or do these regions fold this way also in a full genome context? In other words, is it possible these presumably common elements usually pair much better with other regions of the genome? Without doubt, mFold will take any stretch of 60 bases, random or real, and find cute stems, almost none of which are biologically relevant or energetically optimal. Other than that the preferred seqs can be drawn in this configuration, is there any reason to believe 2D structure plays an actual role in PS recognition here? Again, is induced fit a plausible explanation for the observed RNA density maps? This point should be more strongly emphasized in lns 219-225.

6. The mutational analyses uses TCID50 as the primary parameter of KO phenotypes. Any connection between this and “assembly efficiency” (ln 252) is very speculative. Similar numbers replication centers (not shown) and other referenced infection parameters are just really not convincing that these seqs cause assembly-specific defects.

Minor points:
1. Within the field, “PV” is commonly used to denote “polioviruses” (also very well studied at the structural level) not, as used here, “picornaviruses.” Perhaps there is an abbreviation that would be less confusing?

2. Line 81-82. “PV” even as used here is a family not a genus. Since most viruses in the other picornavirus genera undergo VP0 cleavage, the observations here are likely to be limited to “broad-spectrum anti-viral therapy” extrapolation only within the Parechovirus genus.

3. Move the primer list to Supplementary Info, also genbank list of compared, related viruses.

**Reviewer #2 (Remarks to the Author):**

Shakeel et al provide evidence to extend the packaging signal (PS) concept within viral genomic RNA molecules, developed and largely confirmed for RNA bacteriophages and satellite tobacco necrosis virus, to picornaviruses. They were presented with an excellent example for investigation in human paraecho virus 1 (HpeV1) where high resolution crystallography showed a region of well ordered RNA density, interacting with the capsid protein, that had to have icosahedral symmetry to be visible with the non crystallographic symmetry used for phase determination and refinement. In addition, the fact that Hpe viruses do not undergo a post assembly maturation cleavage event that generates VP4 and VP2 from VP0, like the best studied picornaviruses, argues that such PS may be a part of the larger picornavirus group assembly, but that the interactions are disorganized following the maturation cleavage. This study incorporates a rigorous experimental approach where in vitro analysis of preferred binding sequences to disassembled HpeV1 pentamers is determined by Systematic evolution of ligands by exponential enrichment (SELEX) followed by a bioinformatic analysis of the HpeV1 genome, used for crystallography, for evidence of sequences similar to those selected experimentally. Using robust statistical methods 21 sequences were identified in the genome corresponding to those selected experimentally. The analysis was then expanded to the sequences of HpeV1 strains in Genbank with a significant number of sequences agreeing reasonably well with those selected experimentally and identified in the original HpeV1 genome. They finally derived a consensus sequence GxUxUxxU deemed to be the packaging signal. Analysis of folded sequences for the different genomes suggested that the GxU motif was in a loop and provided the point of contact with the coat protein. Their sequence was then compared to the density for the HpeV1 X-ray structure and they showed that their consensus sequence was a better fit than that used in the published refinement and that their sequence lead to an improved R-factor and allowed a detailed modeling of the protein-RNA interactions. Experimental studies (with appropriate controls) of in vivo assembly were then performed by destroying the RNA consensus sequences while preserving the amino acids encoded. The failure to produce infectious particles in the majority of mutations was striking and dramatically supported the hypothesis. Further, in vitro, studies of pentamer protein interactions with the consensus RNA sequences were performed with demonstrated binding based on micro-scale thermophoresis and EM. Although binding was confirmed, assembly of the pentamers did not take place.

This is an exceptionally comprehensive and revealing study that supports the extension of the PS concept to a novel class of picornaviruses. The authors suggest that the PS assembly model may be appropriate for conventional picornaviruses but is masked by post assembly maturation cleavage. The authors stopped short of looking for the consensus sequence in the myriad of picornavirus genomes that are available, but I suspect that this is underway.

The paper is well written and illustrated with some truly important new insights for virus assembly. The discussion provides a variety of speculations on the details of RNA folding

and its role in the assembly process. The paper is obviously the result of a very refined presentation and, in my opinion, can be published as is.

**Reviewer #3 (Remarks to the Author):**

This manuscript describes the identification of favoured binding sites for CP within the HPeV1 RNA genome. Overall, the methodologies utilized were appropriate, the results were convincing, and the conclusions were reasonable. The main weakness of the work is its similarity to prior studies from the same group reporting a comparable packaging mechanism for bacterial and plant viruses. Nonetheless, this discovery is still significant as: it new for animal viruses, it has potential clinical relevance, and the results will be of interest to a large number of researchers. Suggestions for improvement are provided below.

The study would benefit from a direct measure of virions, rather than the indirect approaches used. Also, the proposed contact sites could be confirmed by cross-linking between the viral RNA and CP.

Ln233, pg11 – briefly explain why mutant design was problematic – and, was codon usage taken into account?

Can the authors provide a possible explanation as to why different effects were observed for the various PS mutants?

What is the relevance of the different affinities of the PSs (i.e. PS affinity does not appear to correlate with importance) and how this feature would integrate into the proposed 5'-to-3' directional packaging proposed in Fig.5?

Minor

Ln32, pg2 - PV is the acronym used specifically for poliovirus, therefore it should not be used to represent the family Picornaviridae, as this could be confusing the readers – just use "picornavirus(es)"

Ln68, pg2 - When first used, the acronym HPeV1 is not defined as Humanparechovirus-1

Ln90, pg5 – "(Supplementary Fig. 1)" seems out of place with respect to the content of this sentence

Fig1a – define arrow in legend Fig2b – define asterisk in legend

Fig2a,b – PS3 is depicted to be in the coding region of VP0 in the diagram – yet it is said to reside in the noncoding region in Ln153, pg7

Fig2d – show the nucleotide identities on the structures so that readers can judge nt positioning without having to decode identity using the colour code. It would also be helpful to highlight the GXUXUX motif within the structures. Same suggestions for Supl. Fig2.

Ln253, pg12 – add “(data not shown)”

Ln269, pg12 – add “(data not shown)”

**Reviewer #4 (Remarks to the Author):**

Human parechovirus-1 contains multiple short regions of ordered RNA density in contact with the virus capsid. The authors show that these sites bind genomic segments according to specific sequence and play an essential role in assembly. Since the binding sites on the coat protein are evolutionarily conserved across the genus, a potential anti-viral target has been identified. Manuscript is well written in most places and results are compelling. Minor issues include occasional need for clarification, especially with regard to use of vague pronouns. Some editing is needed to smooth the overall presentation especially in introduction and discussion.

The abstract does not showcase well the exciting content of the manuscript

Line 48 “The occupancy levels of the density are high, suggesting that they reflect important aspects of the virion structure.” What does “occupancy level” describe? Density magnitude in a map does not necessarily impart function.

Introduction is unclear in several places whether it is RNA density or RNA-capsid interactions that are being described. Example: The assembly and positioning of defined RNA regions at precise locations within these virions suggests that the genome may play an important role(s) in its formation.

Fig 1 is unclear and of poor resolution. In the legend, clarify the “obvious effect on resolution” is visualization of more density corresponding to the RNA genome in the 8.5Å map, and not the higher resolution map. This is clear in the text, but not the legend.

S Fig 1 is too small

Line 213, clarify that the required assembly subunit is what is meant by capsomer, or other modification.

Additional headings may enhance the flow of the manuscript

Fig 4 legend, need citation and explanation of MS2 “TR” and “+++++”

Line 339, please consider omitting “some details are probably incorrect,” to strengthen the paper, as you have made it very clear it is a model based on the data.

By what mechanism might the cleavage of VP0 dislocate most of the PS-CP? What energy or conformational change initiated by VP0 cleavage could possibly result in the dislocation of PS-CP? This seems far-fetched.

Can it be stated as a fact that the PS-CP prevents genome tangles? Or is this supposition?

Line 184, the potential for RNA density to occupy 60 positions within the icosahedral capsid is stated clearly. Less care is taken in other places, such as line 374 that claims there are 60 binding sites.

Line 388, to what is “this” referring?

Methods, for section on SELEX protocol, call figure S1

We thank the reviewers for their insightful comments that have helped us to make significant improvements to the manuscript. Below are our point-by-point responses.

**Reviewer #1 (Remarks to the Author):**

A low resolution cryoEM structure for HPeV1 (8 angs) was supplemented recently with a higher resolution (3.1 angs) X-ray structure, allowing the Aus to create a composite model for the RNA binding sites inside the capsid, visualized as density in the cryoEM structure. This ms describes how that model guided SELEX selection and other experiments on genome sequence aptamers in an attempt to identify relevant RNA packaging signals. The best identified units contain a short consensus motif, GxU, as is consistent with observed densities, structure modeling and sequence variation within this genus of picornaviruses. In total, this is a novel approach, with results likely to influence our understanding of generic assembly processes for RNA viruses. It remains to be seen how far the findings can be extrapolated beyond this particular species of parechoviruses, because other picornaviruses generally undergo extensive internal protein rearrangements and RNA-catalyzed cleavages which surely influence their assembly processes, relative to the simple model proposed here.

The initial statement that the combined EM/X-ray structure guided the SELEX and bioinformatics work allowing us to model an RNA sequence into the EM density (see below) is incorrect. We initiated the SELEX work four years ago on the basis of the intermediate resolution reconstruction of HPeV1. The higher resolution HPeV3 structure that we published in *Nat. Comm*. recently confirms the presence of ordered RNA in another genotype (more than 70% amino acid identity). The HPeV1 X-ray structure came out in late 2015 and showed that a hexanucleotide makes identical contacts to the viral coat proteins in 60 locations within the virion. After further refinement and improvement of this atomic model, we probed whether the principal sequence motif identified via the aptamers within the HPeV1 genome would fit the RNA density. This immediately showed that the interaction was sequence-specificity via hydrogen bonding to amino acid side chains.  The functions of these interactions were then established by reverse genetics by either ablating the recognition motif/secondary structures of the RNA PSs, or by single alanine substitutions of amino acid residues contacting the RNA. We have modified the text at the start of the Results to hopefully make this history clearer, see line 88.

We propose that the conserved RNA-protein contacts are seen in the parechovirus genus, because these viruses do not cleave their VP0 subunits to VP2 and VP4, hence this assembly state is readily visible. In other picornaviruses where VP0 is cleaved, it may also be important, but to our knowledge, no RNA-filled capsid with uncleaved VP0 subunits for such viruses has been reported. Hence our caution in extending our hypothesis to the entire class of viruses.

Points which might strengthen the paper.

1. the SELEX amplification is not particularly impressive ("~400,000 were unique"). Since each of the protomer's protein sequence/structures is identical, isn't this telling you their individual

selection of RNA sequences is actually quite promiscuous and/or involves only very small segments? One might actually interpret Fig 2 as a validation of promiscuity rather than specificity.

We respectfully disagree with this evaluation of the SELEX. Perhaps we were not clear enough in presenting the efficacy of the selection process. The starting (naïve) RNA library contained ~$10^{15}$ distinct sequences (now added at line 104), so obtaining ~4 x $10^5$ unique sequences at the end of the process represents a phenomenal level of selection. This referee perhaps overlooked the nature of the RNA structure-function relationship, which unlike in proteins can easily accommodate multiple nucleotide substitutions with no effect(s) on function. For instance, base-paired regions of a stem can each consist of 4 possible combinations, leading to a potentially functionally neutral library of molecules 10 bp long encompassing over a million distinct sequences.

In fact, despite the sequence diversity of the selected aptamers highlighted by this referee, ~6% of them matched the cognate unique genome sequence with a statistical frequency equivalent to 12 nt identities in a row, a Bernoulli score of 12, that would occur at random at less than one/RNA of the genome's length (added at line 125). This is a statistically robust test, as pointed out by Referee #2. In addition, we asked whether the aptamers "excluded" from further analysis because of this test still had the primary features expected of the proposed PS site. The lowest Mfold free energy folds of all the unique sequences were analysed to see how many fold into a stem-loop containing the GxU motif in the single stranded loop. Over 77% of them do this, confirming that the vast majority were selected on the basis of sequence-specificity for the pentamer target.

2. The Aus assume the interiors of pentamers from disassembled virions (used in the SELEX assays) are identical with regard to assembly competence to native pentamer precursors which have never seen packaged RNA. Is it possible, because of induced fit (see below), these (essentially) pre-trained pentamers might have more restrictive RNA binding references than naive pentamers? Most certainly this would be true for any picornavirus which undergoes VP0 cleavage. Although beyond the scope of the current ms, they may wish to comment on whether baculo-produced or in vitro-produced pentamers might necessarily select the same aptamers. (any pico protomer will make pentamers if properly cleaved by 3C.) That's the essential question here, isn't it? All the discussions about conserved (or not) structure motifs is pretty much hand-waving if the fit primarily relies on induction, nucleated around almost any GxU.

We state that the selection target is pentamer derived from infectious virions, i.e. the selection target has been assembly-competent. From analysis of the X-ray structure of the PS binding site composed of RNA-CP contacts from three VP3 subunits within a pentamer, two of which involve N-terminal arm extensions that reach around the five-fold axis, it seems highly likely that the initial capsomer must also be a pentamer, because protomers lack these RNA binding sites. What the referee says below (pt3) about induced fit of the coat proteins may well apply, but only within a pentamer whose structure is close to that in the virion. The EM structure (Fig 1) shows more of the RNA than the high resolution X-ray structure (as expected). This density is in the form of 5 finger-like extensions approaching the PS-binding region of each pentamer, the lower region of which fits very well when modelled as a base-paired A-type duplex. The existence of such structures that are conserved across the HPeV1 isolates, and even into different genotypes, including the most unrelated, HPeV3 (Figs 6 & S4), suggests this is the way the PS recognition motif is introduced to the binding site initially

(Fig 5), and so the analysis is not moot. The Mfold structures shown through the manuscript illustrate this conformation for the PSs, however the upper region starting from the single-stranded loop sequence must be melted out to make the contacts seen in the X-ray structure, an example of induced fit in the RNA. The detailed RNA-protein contacts may well follow the induced fit ideas described by this Referee, however many PSs contain additional copies of the GxU motif outside the initial loop region, and it is clear from the mutagenesis data that these have no obvious effect on PS function. Consistent with these ideas the NMR solution structure of PS6 is only base paired in the lower stem, i.e. it is consistent with opening up of the upper stem (Varani, *pers comm*.). We have clarified these ideas in the revised manuscript (see line 235 onwards)

The Referee asks us to speculate about non-natively produced pentamers. We acknowledge that picornavirus pentamers can exist in differing states, as exemplified by the formation of empty capsids for bovine enterovirus proteins purified from baculovirus (Li et al, 2012, J.Virol, 86, 13062-12), and icosahedral empty particles during natural infections. Our manuscript concentrates solely on assembly pathways linked to formation of infectious virus.

3. Extensive rearrangement of VP1, VP0 and VP3 protein tails in presence/absence of RNA (i.e. between full/empty particles) is well documented for multiple picornaviruses in both X-ray or cryo-EM structures, regardless of whether the resolved units were self-assembling pre-particles (i.e. never contained RNA), or had been induced to release their RNA (e.g. A-particles). Therefore, the static environment imaged for the interior proteins (current structure) in any full particles (or pentamers) is sure to be quite different than that encountered by the RNA during the assembly process itself. The Fig5 cartoon pretty much ignores the possibility that the RNA/protein fit is mostly likely highly induced, protomer-by-protomer, or RNA motif-by-motif. Were this not the case, there should be 60 nearly equivalent (sequence and 2D structure) RNA motifs, waiting to be strung, unit by unit, into the next available protomer slot. But the observation is, some aptamers are better than others which means the process has to rely on a merely tolerable recognition with the next sequential GxU-like sequence unit deemed capable of induced fit with the next pentamer/protomer. The Aus should acknowledge in Results/Discussion (of their model) that extensive, progressive protein/RNA co-rearrangements are likely to strongly influence the overall assembly process. Their extensive search for commonalities in 2D RNA motifs really becomes moot if this is the case.

The conservation of secondary structures, stem-loops, is not moot because the EM density for the RNA clearly shows a conserved feature, consistent with a stem-loop, below the fragments of icosahedrally-ordered RNA (see above). This is made clear in the new Sup Fig 3. We have explicitly used the term induced fit in the new draft in relation to the unfolding of the uppermost stem of the PS structures shown in Fig 2 (line 241).  The Referee seems to be suggesting that the genome is simply the equivalent of a computer tape that gets caught at successive occurrences of GxU. If that were the case we do not believe the extended density would be seen, since that model for assembly would not require any defined structures either side of the GxU and its three neighbouring bases. See above also for the discussion of additional GxU motifs within PSs. There are 436 occurrences of a GxU triplet in the HPeV1, 71 of which occur in the loops of stem-loops of

the peaks in the Bernoulli plot, i.e. the genome conformation would be scrambled between individual virions if it chose GxU triplets at random (see line 242 onwards).

4. The EM on which key RNA assumptions are based is only at 8.5 angs. This fuzzy density and/or expected G/A first-base were modeled into the 3.1 angs X-ray protein map which itself has poorly resolved RNA densities. Ideally, one would do this exercise using a single much better resolution cryoEM dataset (currently possible at <3.0 angs) instead of superimposed amalgamated manual rebuild. Therefore, the juxtaposition here, while creative, seems to rely on a good deal of unsubstantiated, subjective modeling. Were these results accepted because they fit the Aus expectations?

The electron density for the RNA in the X-ray map at 3.1 Å resolution is well resolved at positions 1 and 3. G and U at these locations, respectively, clearly give the best model in terms of both the fit to the electron density map and interactions with the surrounding protein. The electron density for the bases at the remaining positions is less well defined, with no interactions with the surrounding protein or RNA, other than stacking against the adjacent bases and backbone contacts. Since the phosphate sugar backbone and the base stacking are well resolved in the map at these positions, we interpret this poorer density as being due to base sequence variation at these positions between different sites in the capsid. The EM map shows that these 6 bases form part of a larger, less well ordered or symmetrically arranged RNA structure. There were no prior expectations on the RNA sequence during our structural analysis. Based on rigorous analysis of the X-ray structure alone, we can predict a consensus packaging motif GxU, and this fits perfectly with our SELEX consensus. A 3.1 Å EM reconstruction with icosahedral symmetry applied will still not give a model where the sequence can be read directly beyond the GxU, as it is the application of the symmetry that degrades the signal, whether it is from X-ray or EM. An asymmetric reconstruction could give this if the RNA in every virion has an identical fold, but our experiments in that direction with HPeV3, could not reach atomic resolution with the current computational methods available (Shakeel et al. 2016). This could be because the folds vary, or because the computational methods are not sensitive enough to extract this information.

5. Were the putative RNA 2D motifs returned by mFOLD, as shown in Fig2, Fig6 and S2 all done on local segments (60 b?) of the RNA sequence, or do these regions fold this way also in a full genome context? In other words, is it possible these presumably common elements usually pair much better with other regions of the genome? Without doubt, mFold will take any stretch of 60 bases, random or real, and find cute stems, almost none of which are biologically relevant or energetically optimal. Other than that the preferred seqs can be drawn in this configuration, is there any reason to believe 2D structure plays an actual role in PS recognition here? Again, is induced fit a plausible explanation for the observed RNA density maps? This point should be more strongly emphasized in lns 219-225.

The PS folds were determined by Mfold of 41 nt long fragments, 20 nts either side of the peak in the Bernoulli plot (line 155 onwards). Sup. Fig S2 only shows the folded portions of these fragments which may have been confusing. We now explain this in the legend to that figure. Due to rewriting, the idea of induced fit is now in the paragraph starting at line 235.

6. The mutational analyses uses TCID50 as the primary parameter of KO phenotypes. Any connection between this and “assembly efficiency” (ln 252) is very speculative. Similar numbers replication centers (not shown) and other referenced infection parameters are just really not convincing that these seqs cause assembly-specific defects.

We did not rely solely on the TCID50 results. We checked production of dsRNA, quantitative analysis of genomic RNA, protein translation, and IFA with an antibody specific for viral capsids to have an indication which stage(s) of replication and assembly was affected by the mutations. These analyses were carried out for all of the mutants reported in the paper. The only mutant for which we saw an early effect on the virus life cycle, was PS9M, where the *in vitro* translation of the RNA was not wildtype.  We did not include the IFA data in the paper as they gave no additional information, being consistent with all of the other experiments. As we stated in the results (line 268 onwards) “In order to exclude the possibility that the outcomes are the result of effects on replication, we confirmed that the wild-type and mutants produced similar amounts of viral RNA using real-time PCR. Further, immunofluorescence with an anti-dsRNA antibody showed that there are also similar numbers of viral replication centres for all mutants in all the cells used. All the mutant genomes tested (except PS9-M) also produce similar *in vitro* translation products. Hence, in PS21, PS14 and PS18, the decreases in viral titre are most likely the result of alterations in assembly efficiency (Table 2)”.

Minor points:
1. Within the field, “PV” is commonly used to denote “polioviruses” (also very well studied at the structural level) not, as used here, “picornaviruses.” Perhaps there is an abbreviation that would be less confusing?

We have replaced all instances of “PV(s)” in our text with the word(s) picornavirus(es) to avoid this confusion.

2. Line 81-82. “PV” even as used here is a family not a genus. Since most viruses in the other picornavirus genera undergo VP0 cleavage, the observations here are likely to be limited to “broad-spectrum anti-viral therapy” extrapolation only within the Parechovirus genus.

We replaced PV with Parechovirus in the stated lines.

3. Move the primer list to Supplementary Info, also genbank list of compared, related viruses.

The primer list and Genbank ids have been moved to the Supplemental Information.

**Reviewer #2 (Remarks to the Author):**

Shakeel et al provide evidence to extend the packaging signal (PS) concept within viral genomic RNA molecules, developed and largely confirmed for RNA bacteriophages and satellite tobacco necrosis virus, to picornaviruses. They were presented with an excellent example for investigation in human paraecho virus 1 (HpeV1) where high resolution crystallography showed a region of well ordered RNA density, interacting with the capsid protein, that had to have icosahedral symmetry to be visible with the non crystallographic symmetry used for phase determination and refinement. In addition, the fact that Hpe viruses do not undergo a post assembly maturation cleavage event that generates VP4 and VP2 from VP0, like the best studied picornaviruses, argues that such PS may be a part of the larger picornavirus group assembly, but that the interactions are disorganized following the maturation cleavage. This study incorporates a rigorous experimental approach where in vitro analysis of
preferred binding sequences to disassembled HpeV1 pentamers is determined by Systematic evolution of ligands by exponential enrichment (SELEX) followed by a bioinformatic analysis of the HpeV1 genome, used for crystallography, for evidence of sequences similar to those selected experimentally. Using robust statistical methods 21 sequences were identified in the genome corresponding to those selected experimentally. The analysis was then expanded to the sequences of HpeV1 strains in Genbank with a significant number of sequences agreeing reasonably well with those selected experimentally and identified in the original HpeV1 genome. They finally derived a consensus sequence GxUxUxxU deemed to be the packaging signal. Analysis of folded sequences for the different genomes suggested that the GxU motif was in a loop and provided the point of contact with the coat protein. Their sequence was then compared to the density for the HpeV1 X-ray structure and they showed that their consensus
sequence was a better fit than that used in the published refinement and that their sequence lead to an improved R-factor and allowed a detailed modeling of the protein-RNA interactions. Experimental studies (with appropriate controls) of in vivo assembly were then performed by destroying the RNA consensus sequences while preserving the amino acids encoded. The failure to produce infectious particles in the majority of mutations was striking and dramatically supported the hypothesis. Further, in vitro, studies of pentamer protein interactions with the consensus RNA sequences were performed with demonstrated binding based on micro-scale thermophoresis and EM. Although binding was confirmed, assembly of the pentamers did not take place.

This is an exceptionally comprehensive and revealing study that supports the extension of the PS concept to a novel class of picornaviruses. The authors suggest that the PS assembly model may be appropriate for conventional picornaviruses but is masked by post assembly maturation cleavage. The authors stopped short of looking for the consensus sequence in the myriad of picornavirus

genomes that are available, but I suspect that this is underway.

The paper is well written and illustrated with some truly important new insights for virus assembly. The discussion provides a variety of speculations on the details of RNA folding and its role in the assembly process. The paper is obviously the result of a very refined presentation and, in my opinion, can be published as is.

We welcome these very positive comments.

**Reviewer #3 (Remarks to the Author):**

This manuscript describes the identification of favoured binding sites for CP within the HPeV1 RNA genome. Overall, the methodologies utilized were appropriate, the results were convincing, and the conclusions were reasonable. The main weakness of the work is its similarity to prior studies from the same group reporting a comparable packaging mechanism for bacterial and plant viruses. Nonetheless, this discovery is still significant as: it new for animal viruses, it has potential clinical relevance, and the results will be of interest to a large number of researchers. Suggestions for improvement are provided below.

We find that the similarity to the mechanism we have described previously in relation to bacterial and plant viruses (see below) is actually a major strength of the present work. It explains what until now has been a complete mystery, namely the genome packaging specificity of at least one genus of the picornaviruses. It also suggests that there is much more commonality between viral families than has been recognised previously.

The study would benefit from a direct measure of virions, rather than the indirect approaches used. Also, the proposed contact sites could be confirmed by cross-linking between the viral RNA and CP.

The viral titration is a direct measure of virion number.

We have previously performed a reversible cross-linking–peptide fingerprinting (RCAP) analysis by formaldehyde cross-linking on bacteriophage MS2 (Rolfsson et al, JMB, 2016, 428, 431-48). It confirmed most of the proposed PS-CP contact sites in that virion, but did not narrow down the RNA contact sites on the proteins significantly. These types of analysis require very large quantities of viral material (hence high titre bacteriophage are suitable, but not low titre human viruses) and would take up to 12 months of additional work. As a result we think it is unreasonable to be asked to undertake a similar analysis here, given the small quantities of virus that are available, and an X-ray density map showing the RNA-CP contacts.

Ln233, pg11 – briefly explain why mutant design was problematic – and, was codon usage taken into account?

We wanted to introduce silent mutations within the PS6, PS7 and PS14, however due to the way the reading frame crosses the GxU consensus motif and the amino acids encoded which have few

codon options, simple synonymous mutations fail to change the recognition motif. Therefore, we introduced silent mutations in the flanking regions of these PSs, i.e. along the stem of the structures shown in Fig 2, which did disrupted the recognition motif in the PSs by forcing it to basepair with other areas of the genome. We have rephrased the section, taking away the “problematic” to make it clearer. (Line 252 onwards).

We did not account for codon usage while introducing silent mutations as our sole aim was to disrupt the secondary structure of the PSs.

Can the authors provide a possible explanation as to why different effects were observed for the various PS mutants?
What is the relevance of the different affinities of the PSs (i.e. PS affinity does not appear to correlate with importance) and how this feature would integrate into the proposed 5’-to-3’ directional packaging proposed in Fig.5?

We propose in the manuscript (line 729 onwards) that PSs are introduced to pentamers via the 2C-protomer complex (Fig 5), binding of PSs to a pentamer being completed before movement to a neighbouring pentamer. Disrupted PSs are thus part of a co-operative assembly process and individual mutant sites can be expected to have different levels of effect from zero to lethality depending on their neighbouring PSs. We have added this to the discussion (Line 362 onwards)

We have shown previously that different PS affinities are important for the efficiency of the assembly process. In particular, PS affinity distributions direct the assembly process along specific capsid assembly pathways. In analogy to this, we expect the PS distribution in HPeV1 to favour specific assembly pathways (i.e. determine the order in which pentamers are added to the growing capsid shell).

Minor

Ln32, pg2 - PV is the acronym used specifically for poliovirus, therefore it should not be used to represent the family Picornaviridae, as this could be confusing the readers – just use “picornavirus(es)”

We thank the reviewer for pointing out the wrong usage of the acronym. We have replaced all instances of “PV(s)” with picornavirus(es).

Ln68, pg2 - When first used, the acronym HPeV1 is not defined as Humanparechovirus-1

We have defined the acronym HPeV1 where it is first used (Line 42).

Ln90, pg5 – “(Supplementary Fig. 1)” seems out of place with respect to the content of this sentence

We have removed “(Supplementary Fig. 1)” from this line and placed it in the sentence where it is referred correctly (Line 94).

Fig1a – define arrow in legend Fig2b – define asterisk in legend

We have defined the arrow in the Supplementary Fig 1a. The sentence in the figure legends now reads as “A thermofluor assay was used to identify the temperature at which the capsid destabilizes using Sybr Safe DNA dye (Invitrogen) which fluoresces upon binding to the RNA. Arrow indicates the temperature at which RNA becomes accessible to the dye”.

To define the asterisks in Fig 2a and Fig 2b we have added the following sentences “The asterisks in (a) and (b) indicate two peaks named PS8A and PS8B, which are below the statistical cut-off but are highly conserved as significant peaks in >50% of the strains other than Harris. In fact their potential secondary structures (Supplementary Fig. 2b) have the same conserved sequence motif. Hence they can be additional putative PSs” (Line 684 onwards).

Fig2a,b – PS3 is depicted to be in the coding region of VP0 in the diagram – yet it is said to reside in the noncoding region in Ln153, pg7

We thank the reviewer for pointing out this mistake. The PS3 is indeed in the VP0 coding region. We have deleted the sentence where it is said to reside in the noncoding region.

Fig2d – show the nucleotide identities on the structures so that readers can judge nt positioning without having to decode identity using the colour code. It would also be helpful to highlight the GXUXUX motif within the structures. Same suggestions for Supl. Fig2.

These have now been included in both figures. In particular, the start of the GxU motif has been indicated by an arrow pointing to the G.

Ln253, pg12 – add “(data not shown)”

We refer to Table 2 because we have summarized the results there.

Ln269, pg12 – add “(data not shown)”

We refer to Table 2 because we have summarized the results there.

**Reviewer #4 (Remarks to the Author):**

Human parechovirus-1 contains multiple short regions of ordered RNA density in contact with the virus capsid. The authors show that these sites bind genomic segments according to specific sequence and play an essential role in assembly. Since the binding sites on the coat protein are evolutionarily conserved across the genus, a potential anti-viral target has been identified. Manuscript is well written in most places and results are compelling. Minor issues include occasional need for clarification, especially with regard to use of vague pronouns. Some editing is needed to smooth the overall presentation especially in introduction and discussion.

The abstract does not showcase well the exciting content of the manuscript

We have rewritten the Abstract so that hopefully it addresses this concern.

Line 48 “The occupancy levels of the density are high, suggesting that they reflect important aspects of the virion structure.” What does “occupancy level” describe? Density magnitude in a map does not necessarily impart function.

Here, high occupancy level refers to the fact that the electron density levels for portions of the RNA contacting the capsid are as high, or almost as high, as in the protein capsid. This shows that these portions of the RNA genome are present in all, or nearly all, copies of the icosahedral asymmetric unit. Therefore, it is fair to regard these portions of the RNA genome as a structural component of the capsid, of equal importance to any other component.

Introduction is unclear in several places whether it is RNA density or RNA-capsid interactions that are being described. Example: The assembly and positioning of defined RNA regions at precise locations within these virions suggests that the genome may play an important role(s) in its formation.

We have rephrased several sentences in the introduction section to make a clear distinction between RNA density and RNA-capsid interactions. These rephrased sentences are as follow:

“All these virions contain 60 RNA fragments that make similar contacts with their coat proteins around the five-fold vertices. The occupancy levels of the RNA densities are high, suggesting that they reflect important aspects of the virion structure” (Line 45 onwards).

“The role(s) of this RNA density, however, requires a functional explanation, especially as these structures may represent an earlier evolutionary state of the wider group of picornaviruses.The assembly and positioning of defined RNA fragments at precise locations within these virions suggests that the genome may play an important role(s) in the virus assembly” (Line 49 onwards).

“The classical PS hypothesis assumes a single PS site (a specific RNA fragment) with affinity for cognate coat protein (CP) that results in formation of an RNA-CP assembly initiation complex”. (Line 56 onwards)

Fig 1 is unclear and of poor resolution. In the legend, clarify the “obvious effect on resolution” is visualization of more density corresponding to the RNA genome in the 8.5Å map, and not the higher resolution map. This is clear in the text, but not the legend.

The original figure is of high resolution, complies with Journal guidelines and when printed is sharp. When we look at the combined pdf and the smaller versions of the figures on certain screens it does appears fuzzy, but we think that there is no serious issue with the current figure (see comment below also). We have rephrased the sentence in the Fig. 1 legend as follows to clarify the point raised above by the reviewer (Lines 665-668).

S Fig 1 is too small

Again the submitted figure complied with the guidelines and in our documents is an entire page. We believe this may be an issue with the on-line system and what the Referee could download.

Line 213, clarify that the required assembly subunit is what is meant by capsomer, or other modification.

For clarification we have defined the meaning of capsomer in the first instance where it is used. The sentence reads as follows “In order to explore whether the ordered RNA densities within the new picornaviruses structures correspond to such PSs, we isolated aptamers against the pentameric HPeV1 capsomer (the assembly subunit required to make the full capsid)” (Lines 66).

Additional headings may enhance the flow of the manuscript

The word limit precludes our doing this.

Fig 4 legend, need citation and explanation of MS2 “TR” and “+++++”

We have provided the MS2 TR “+++++” explanation in the following sentence “These were compared to a known PS-CP interaction, that of the 19 nt bacteriophage MS2 TR (transcriptional operator) stem-loop and its CP[45] (Kd ~1-4 nM, depending on binding assay used), which was designated as +++++, i.e. low nano-molar affinity.” (Lines 722-724).

Line 339, please consider omitting “some details are probably incorrect,” to strengthen the paper, as you have made it very clear it is a model based on the data.

We have deleted it as suggested.

By what mechanism might the cleavage of VP0 dislocate most of the PS-CP? What energy or conformational change initiated by VP0 cleavage could possibly result in the dislocation of PS-CP? This seems far-fetched.

Comparison of virion versus provirion (empty capsid), for example, in poliovirus (Basavappa *et al* Protein Science 1994; Hogle *et al* Science 1985) shows spatial rearrangement of VP4 and VP2 following cleavage of VP0 . This autocatalytic serine protease-type cleavage is hypothesized to require encapsidated viral RNA (Arnold *et al* PNAS 1987). Such coat protein movements within the context of the virion could easily result in the loss of the RNA-CP contacts used during assembly. Whatever the details of the VP0 cleavage in other picornaviruses, if they share a similar chain of protein contacts to RNA packaging signals, the protein conformational changes induced by cleavage are very likely to lead to conformational changes within the N-terminal tails of VP3 destroying the RNA-binding sites seen for the parechoviruses.

Can it be stated as a fact that the PS-CP prevents genome tangles? Or is this supposition?

There is no experimental evidence showing the PS-CP prevents genome tangling. We have removed this suggestion.

Line 184, the potential for RNA density to occupy 60 positions within the icosahedral capsid is stated clearly. Less care is taken in other places, such as line 374 that claims there are 60 binding sites.

We have rephrased the sentence, it now reads as follows “The numbers are consistent with the 60 potential RNA-binding sites seen in Fig. 1” (Line 376).

Line 388, to what is “this” referring?

We have replaced “this” with “the above analysis of the selected HPeV1 aptamer library to identify putative PSs in other sequence-conserved parechoviruses” (Lines 391).

Methods, for section on SELEX protocol, call figure S1

Added the following “(see Supplementary Fig. 1 for more details)” in line 415 of the Method section on SELEX protocol.

## REVIEWERS' COMMENTS:

**Reviewer #1 (Remarks to the Author):**

The authors have responded to previous reviews with clarifying information and edits. While some of their their interpretation of the results may still be somewhat debatable, the manuscript, as now presented is suitable for publication.

**Reviewer #3 (Remarks to the Author):**

The authors have adequately addressed the concerns of this reviewer. Consequently, the revised manuscript is suitable for publication.

**Reviewer #4 (Remarks to the Author):**

Comments have been adequately addressed.
